# Circulating Levels of Interferon Regulatory Factor-5 Associates With Subgroups of Systemic Lupus Erythematosus Patients

**DOI:** 10.3389/fimmu.2019.01029

**Published:** 2019-05-17

**Authors:** Helena Idborg, Arash Zandian, Elena Ossipova, Edvard Wigren, Charlotta Preger, Fariborz Mobarrez, Antonio Checa, Azita Sohrabian, Pascal Pucholt, Johanna K. Sandling, Cátia Fernandes-Cerqueira, Johan Rönnelid, Vilija Oke, Giorgia Grosso, Marika Kvarnström, Anders Larsson, Craig E. Wheelock, Ann-Christine Syvänen, Lars Rönnblom, Kim Kultima, Helena Persson, Susanne Gräslund, Iva Gunnarsson, Peter Nilsson, Elisabet Svenungsson, Per-Johan Jakobsson

**Affiliations:** ^1^Division of Rheumatology, Department of Medicine Solna, Karolinska Institutet, Karolinska University Hospital, Stockholm, Sweden; ^2^SciLifeLab, Division of Affinity Proteomics, Department of Protein Science, KTH Royal Institute of Technology, Stockholm, Sweden; ^3^Department of Medical Sciences, Akademiska Hospital, Uppsala University, Uppsala, Sweden; ^4^Division of Physiological Chemistry 2, Department of Medical Biochemistry and Biophysics, Karolinska Institutet, Stockholm, Sweden; ^5^Department of Immunology, Genetics and Pathology, Uppsala University, Uppsala, Sweden; ^6^Department of Medical Sciences, Rheumatology, Uppsala University, Uppsala, Sweden; ^7^Department of Medical Sciences, Clinical Chemistry, Uppsala University, Uppsala, Sweden; ^8^Department of Medical Sciences, Molecular Medicine and Science for Life Laboratory, Uppsala University, Uppsala, Sweden; ^9^Science for Life Laboratory, Drug Discovery and Development & School of Engineering Sciences in Chemistry, Biotechnology and Health, KTH Royal Institute of Technology, Stockholm, Sweden

**Keywords:** Interferon regulating factor 5 (IRF5), antibody suspension bead arrays, subgroups, biomarker discovery, plasma proteomics, unsupervised clustering, hierarchical clustering, SLE - Systemic Lupus Erythematous

## Abstract

Systemic Lupus Erythematosus (SLE) is a heterogeneous autoimmune disease, which currently lacks specific diagnostic biomarkers. The diversity within the patients obstructs clinical trials but may also reflect differences in underlying pathogenesis. Our objective was to obtain protein profiles to identify potential general biomarkers of SLE and to determine molecular subgroups within SLE for patient stratification. Plasma samples from a cross-sectional study of well-characterized SLE patients (*n* = 379) and matched population controls (*n* = 316) were analyzed by antibody suspension bead array targeting 281 proteins. To investigate the differences between SLE and controls, Mann–Whitney *U*-test with Bonferroni correction, generalized linear modeling and receiver operating characteristics (ROC) analysis were performed. K-means clustering was used to identify molecular SLE subgroups. We identified Interferon regulating factor 5 (IRF5), solute carrier family 22 member 2 (SLC22A2) and S100 calcium binding protein A12 (S100A12) as the three proteins with the largest fold change between SLE patients and controls (SLE/Control = 1.4, 1.4, and 1.2 respectively). The lowest *p*-values comparing SLE patients and controls were obtained for S100A12, Matrix metalloproteinase-1 (MMP1) and SLC22A2 (p_adjusted_ = 3 × 10^−9^, 3 × 10^−6^, and 5 × 10^−6^ respectively). In a set of 15 potential biomarkers differentiating SLE patients and controls, two of the proteins were transcription factors, i.e., IRF5 and SAM pointed domain containing ETS transcription factor (SPDEF). IRF5 was up-regulated while SPDEF was found to be down-regulated in SLE patients. Unsupervised clustering of all investigated proteins identified three molecular subgroups among SLE patients, characterized by (1) high levels of rheumatoid factor-IgM, (2) low IRF5, and (3) high IRF5. IRF5 expressing microparticles were analyzed by flow cytometry in a subset of patients to confirm the presence of IRF5 in plasma and detection of extracellular IRF5 was further confirmed by immunoprecipitation-mass spectrometry (IP-MS). Interestingly IRF5, a known genetic risk factor for SLE, was detected extracellularly and suggested by unsupervised clustering analysis to differentiate between SLE subgroups. Our results imply a set of circulating molecules as markers of possible pathogenic importance in SLE. We believe that these findings could be of relevance for understanding the pathogenesis and diversity of SLE, as well as for selection of patients in clinical trials.

## Introduction

Systemic Lupus Erythematosus (SLE) is a heterogeneous systemic autoimmune disorder with a plethora of clinical manifestations. Clinical and immunological criteria, defined by the American College of Rheumatology (ACR) (1), are used to classify the disease for research purposes, but reliable diagnostic biomarkers are lacking. The diversity of the disease is a great obstacle and might reflect differences in pathogenesis between different subgroups. Several recent reviews highlight the importance of defining subgroups of SLE to better treat patients with tailored medicine, and in order to increase efficacy in clinical trials (2–5). Accordingly, there is a great need for exploring subgrouping and novel diagnostic biomarkers in SLE.

Few biomarkers have been implemented in clinical routine reflecting the difficulties of biomarker research in lupus (6). Screening of a large number of proteins (>50) but in a limited number (<50) of SLE patients have been performed to identify biomarkers in SLE (7–10). In this study we analyzed 281 proteins using a suspension bead affinity proteomics approach (11), in plasma samples from a total of 695 individuals comprising SLE and matched controls. Selection of proteins is crucial to obtain representative protein profiles. However, the current knowledge of protein functions is far from complete and transcription factors and other nuclear molecules could have unknown functions in the circulation or may, regardless of function, constitute novel biomarkers. The intra- and extracellular functions of a protein might be different and unconventional secretion is also possible (12). Therefore, both nuclear and cytoplasmic molecules are relevant to study in the circulation with the aim to identify potential biomarkers and possible pathogenic pathways.

In a previous study we presented protein profiles for two predefined SLE subgroups, delineated based exclusively on the autoantibody profiles, but also corresponding to clinical observations and experience (13). In the present study we used a different approach and performed unsupervised clustering of the obtained protein profiles to investigate an unprejudiced division of SLE patients. In addition, experimental validation of biomarker candidates discriminating between SLE and control was performed. Our main focus was to identify molecular subgroups in SLE since these, despite similar clinical phenotypes, may benefit from different treatment perspectives.

## Materials and Methods

**Figure 1:**
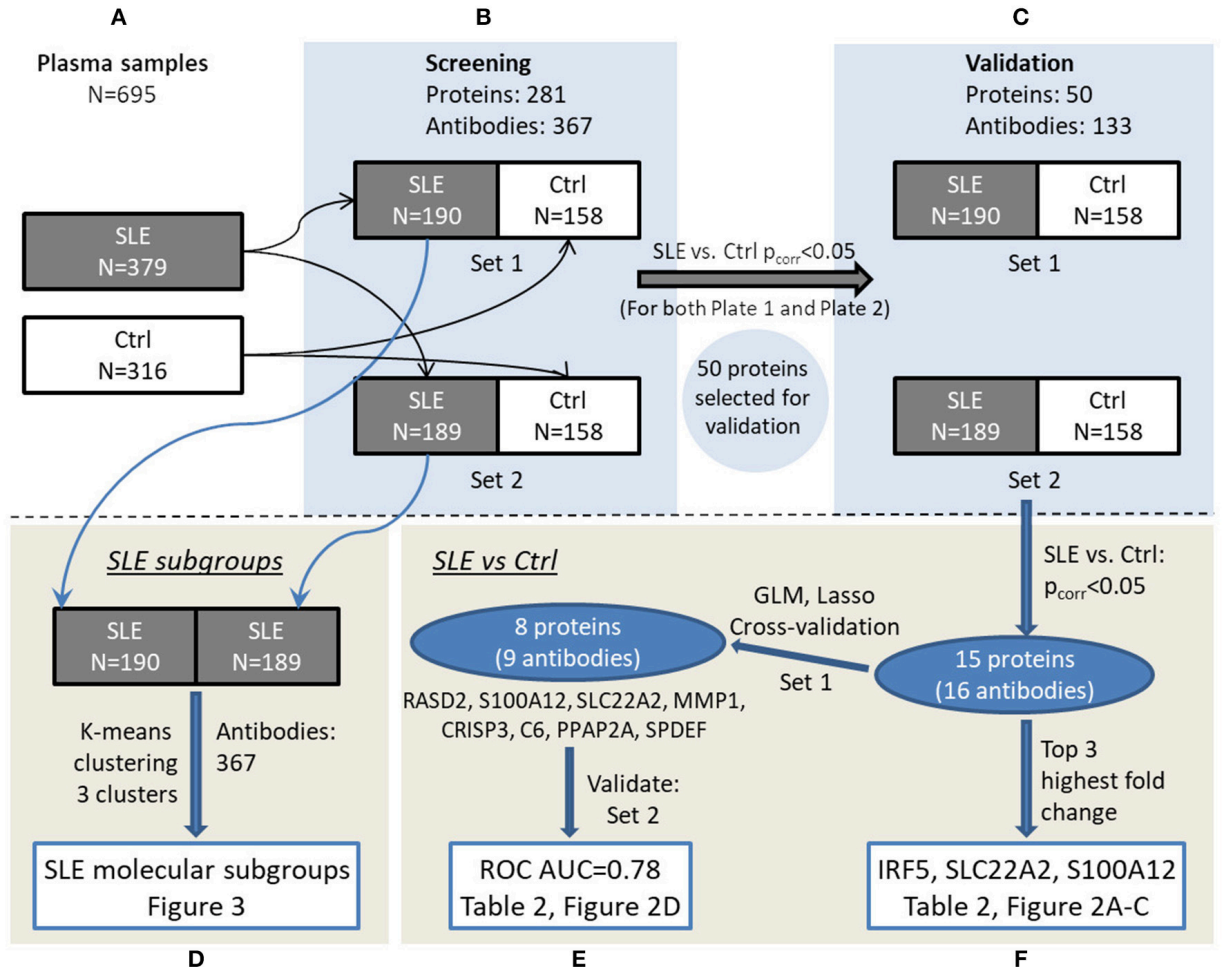
Overview of the experimental workflow. Plasma samples **(A)** were randomized in set 1 and set 2 for screening phase **(B)** followed by validation phase **(C)**. Data were analyzed to investigate SLE subgroups **(D)** as well as comparing SLE and control in a multivariate **(E)** and univariate **(F)** manner, respectively, and main results can be viewed in the referred figures.

Plasma protein profiles were obtained for SLE patients and controls utilizing antibody suspension bead arrays for protein profiling. An overview of the study design can be found in Figure 1.

### Patient Cohort and Controls

Fasting plasma samples were obtained from patients in the Karolinska SLE cohort consisting of 379 SLE patients and 316 population-based controls with matching age, gender and residential area. All SLE patients included in this cross-sectional study, were adults and diagnosed according to the ACR SLE criteria (1). Both patients and controls underwent a structured interview and physical examination as previously described (14). Clinical and serological data for the SLE patients are summarized in Table 1 and in previous work (13). Medication is reported in Supplementary Table S-1, and demographic data for the controls are shown in Supplementary Table S-2.

**Table 1:** Clinical and serological data are reported for the three molecular subgroups as well as for the entire cohort of SLE patients.

	**Entire SLE cohort^a^**	**Molecular SLE subgroups^a^**			**Comparing SLE subgroups^b^**		
	***n* = 357**	**RF-IgM/SSA/SSB subgroup *N* = 51**	**IRF5 low subgroup *N* = 129**	**IRF5 high subgroup *N* = 177**	**RF-IgM/SSA/SSB vs. IRF5 low**	**RF-IgM/SSA/SSB vs. IRF5 high**	**IRF5 low vs. IRF5 high**
Age (years)	47.2 (34.3–58.1)	45.4 (33.6–56.8)	41.4 (31.1–54.2)	51.0 (37.8–60.3)	*P* = 0.50	*P* = 0.07	***p*** **=** **0.0003**
Gender %F	87%	90%	87%	86%	0.62	0.65	0.92
Disease duration (years)	11.5 (4.4–21.7)	6.9 (1.5–14.4)	11.6 (4.5–20.6)	12.9 (5.3–23.2)	*P* = 0.06	*P* = 0.005	*P* = 0.19
SLE ACR criteria	6 (5–7)	6 (5–7)	6 (5–7)	6 (5–7)	*P* = 0.81	*P* = 0.43	*P* = 0.18
SLAM	6 (4–10)	8 (5–12)	6 (3.5–9.5)	6 (3.5–9.5)	*P* = 0.02	*P* = 0.02	*P* = 0.90
SLEDAI-2k	4 (0–7)	4 (1–7)	3 (0.5–7.5)	4 (0–7)	*P* = 0.74	*P* = 0.70	*P* = 0.96
C3a *Kruskal–Wallis test p*<*0.0001*	268.4 (192.7–537.1)	351.8 (243.2–991.4)	434.8 (181.7–3092)	250.9 (191.3–324.2)	*P* = 0.83	***P*** **<** **0.0001**	***P*** **<** **0.0001**
RF IgA (IU/ml) *Kruskal–Wallis test p*<*0.0001*	5.3 (3.4–12)	16.6 (5.6–66.9)	4.1 (2.8–7.3)	5.7 (3.7–11.2)	***P*** **<** **0.0001**	***P*** = **0.0001**	***P*** = **0.0005**
RF IgG (μg/ml) *Kruskal–Wallis test p = 0.0001*	11 (6.9–23)	20 (8.9–54.6)	10 (6.5–17.5)	11 (6.8–19.2)	***P*** **<** **0.0001**	***P*** = **0.0004**	*P* = 0.23
RF IgM (IU/ml) *Kruskal–Wallis test p*<*0.0001*	1.3 (0.63–4.7)	28 (13.5–44.6)	1.1 (0.6–2.4)	1.1 (0.5–2.1)	***P*** **<** **0.0001**	***P*** **<** **0.0001**	*P* = 0.67
IgA total (mg/ml)	2.8 (2–3.9)	3.1 (2.1–4.2)	2.7 (1.9–3.6)	2.9 (2–3.9)	*P* = 0.08	0.38	*P* = 0.15
IgG total (mg/ml)	12.8 (10.4–16.6)	16.7 (12.7–20.6)	11.7 (9.5–14.7)	12.8 (10.3–16.1)	***P*** **<** **0.0001**	***P*** **<** **0.0001**	*P* = 0.05
IgM total (mg/ml)	0.92 (0.58–1.4)	1.2 (0.92–2.1)	0.96 (0.62–1.40)	0.8 (0.49–1.3)	*P* = 0.0017	***P*** **<** **0.0001**	*P* = 0.03
ESR (mm/hour)	19 (11–33)	30 (16.5–46)	14 (9–27)	21 (12–36)	***P*** **<** **0.0001**	*P* = 0.04	***P*** = **0.0004**
hsCRP (mg/l)	1.7 (0.68–5.3)	1.4 (0.51–5.7)	1.1 (0.48–4.7)	2.2 (0.83–5.8)	*P* = 0.28	*P* = 0.18	***P*** = **0.0003**
Fibrinogen (g/l)	4.1 (3.4–5.0)	3.9 (3.1–4.6)	3.8 (3.2–4.8)	4.4 (3.6–5.2)	*P* = 0.95	*P* = 0.006	***P*** = **0.0005**
TNF-α (pg/ml)	4.5 (3.3–6.2)	4.8 (3.5–6.7)	4.0 (2.8–5.7)	5.1 (3.6–6.4)	*P* = 0.015	*P* = 0.77	***P*** = **0.0005**
Fibronectin (mg/ml) *Kruskal–Wallis test p = 0.0008*	0.38 (0.25–0.46)	0.40 (0.29–0.50) *N = 33*	0.41 (0.32–0.48) *N = 80*	0.31 (0.19–0.44)*N = 95*	*P* = 0.78	*P* = 0.03	***P*** = **0.0002**
Leptin (mg/ml) *Kruskal–Wallis test p = 0.0002*	14294 (4776–27938)	13321 (5026–21162)	7878 (2240–20389)	19502 (8474–48617)	*P* = 0.23	*P* = 0.05	***P*** **<** **0.0001**

*SLE American College of Rheumatology (ACR) classification criteria; SLAM, SLE Activity Measure (15); SLEDAI-2K, SLE Disease Activity Index (16). C3, Complement factor 3, RF IgA/G/M, Rheumatoid factor immunoglobulin A/G/M; ERS, erythrocyte sedimentation rate; hsCRP, high sensitivity C-reactive protein*,

a *Median (25% quantile - 75% quantile), NR, not reported. Serology data obtained as described in previous work*.

b *Mann–Whitney U-test for pairwise comparison of subgroups was used to characterize subgroups. P-values <0.001 without adjustment for multiple testing are highlighted in bold. Kruskal–Wallist test, i.e., comparing more than two groups and compensating for multiple testing, highlighted only RF-IgM, RF-IgG, RF-IgA, Leptin, Fibronectin and C3a as significantly different (names highlighted in italic)*.

### Protein Profiling by Antibody Suspension Bead Arrays

A number of 281 proteins were selected as previously described (13), i.e., based on published data on suggested biomarkers in inflammation/SLE/myositis, microarray data comparing SLE and controls and an untargeted mass spectrometry-based proteomic analysis suggesting additional biomarker candidates. A customized set of 367 antibodies (Supplementary Table S-3) was utilized to target unique epitopes of these proteins in a screening experiment (Figure 1B) (13). The antibodies were selected from the Human Protein Atlas (HPA, www.proteinatlas.org) project and are affinity-purified polyclonal antibodies that have been extensively validated (17). Protein profiles were generated using antibody suspension bead array (18). In brief, the 367 HPA antibodies were attached to color-coded magnetic beads, then incubated with 45 μl diluted and biotinylated EDTA-plasma, followed by an addition of streptavidin-conjugated R-phycoerythrin (Invitrogen), and finally analyzed using a FlexMap3D instrument (Luminex Corp.). Data was evaluated as described below and 50 proteins (53 antibodies) were selected for further validation experiments (Figure 1C). In the validation experiment, additional HPA antibodies (*n* = 80) targeting other antigenic regions of these proteins were coupled to beads resulting in a validation assay of 133 antibodies toward the selected 50 proteins (Supplementary Table S-4).

### Data Analysis of Antibody Suspension Bead Array Data

The measured signals, reported as median fluorescent intensities (MFI) from FlexMap3D were imported into R (19). As previously described (20), outliers were identified in the raw data by robust principal component analysis (R package: rrcov) and excluded from further analysis. Subsequently, probabilistic quotient normalization (PQN) was performed on the MFIs to compensate for dilution errors and/or total amount of plasma proteins of the samples (21), followed by LOESS normalization on MA coordinates, per antibody, based on the MFIs to minimize the batch effects (22). Data quality was assessed by comparing replicates per 96-well plate, in combined 384-well plates and inter 384-well plates. Thereafter the data was split into two separate but comparable datasets (Figure 1B) with similar age and gender distribution and equal number of SLE patients and controls (Supplementary Table S-2). Set 1 consisted of 190 SLE patients and 158 controls, and set 2 of 189 SLE patients and 158 controls. This data is referred to as the data from the screening phase. Proteins reaching significance (after Bonferroni correction) comparing SLE and control, with the same direction in fold change between SLE/control, in both sample set 1 and set 2 in screening phase, were selected for validation (Figure 1C, *n* = 50). The validated proteins that were significantly different comparing SLE and controls (*n* = 15), were used for further interpretation. A generalized linear model with lasso regularization (R package: glmnet) was used to find panels of proteins to predict SLE patients and controls where the sample set 1 and set 2 corresponded to test set and training set, respectively. This was followed by analysis and visualization by performing receiver operating characteristic (ROC) analysis (R package: pROC) and confidence intervals (CI) for the area under the curve (AUC) were calculated (23).

In order to identify molecular SLE subgroups, unsupervised clustering was applied on the screening data. To prepare the data for principal component analysis (PCA), the data for each dataset (190 SLE patients in set 1 and 189 SLE patients in set 2) was log2-transformed and centered on the mean of each antibody. In set 1 PC1 and PC2 explained 14 and 12% respectively of the variance, and in set 2 the explained variances by PC1 and PC2 were 18 and 16% respectively. Clustering of samples was done on the first two principal components by using K-means clustering, emphasizing on the variables with greatest variance and the Calinski-Harabasz criterion was used to find the number of clusters in the data.

### Production of Recombinant IRF5 Protein

Multiple constructs of IRF5 (Uniprot ID Q13568) were sub-cloned into the expression vectors pNIC28-Bsa4 and pNIC-Bio3 (Genbank acc. No EF198106, JN792439). After performing small-scale screening for soluble recombinant protein expression as previously described (24), clones corresponding to constructs covering the regions M1-V120 and E232-L434 were selected for generation of single-chain fragment variable (scFv) binders. Expression and purification of selected clones and full-length IRF5 was performed essentially as previously described (25, 26), and a detailed protocol can be found in Supplementary Methods and Results. Final protein batches were analyzed by SDS-PAGE and subsequently flash frozen in liquid nitrogen and stored at −80°C until use.

### Generation of Antibody Fragments Against IRF5

Single-chain fragment variable (scFv) clone J-IRF5-5 was generated by phage display technology using a human synthetic library denoted SciLifeLib. The phage selection procedure was performed basically as described earlier (27), but the first round of selection, including the steps of antigen-phage incubation to trypsin elution, was carried out in 1.5 ml tubes on a rotator with no automation. The number of washing steps was modified and increased with succeeding selection rounds; five in round one and seven in round four. Also, the recovered phages were propagated in XL1-Blue *E. coli* between the selection rounds. Re-cloning of the selected material in pool followed by transformation into TOP10 *E. coli*, small-scale expression of 94 randomly picked scFv and subsequent enzyme-linked immunosorbent assay (ELISA) for detection of recombinant full-length IRF5, i.e., verifying binding to target, and sequencing experiments were performed equivalent to previously reported (27). Affinity measurements were performed using a Biacore T200 biosensor instrument (GE Healthcare) as described in Supplementary Figure S-1. The top candidate (J-IRF5-5), binding the construct region E232-L434 of IRF5, obtained a measured affinity of 5 nM. Validation by ELISA and Biacore was extended by using IP-MS performed on cell lysate from HEK293 cells (300 μl) as previously described (27). The lysate was spiked with a small amount of recombinant IRF5 full-length protein (0.7 μg), as IRF5 is normally expressed at very low levels in HEK293 cells, and MS data was acquired in data dependent mode using a top 10 method. IRF5 was identified as the highest ranked protein in the obtained list of proteins (Supplementary Table S-5). This verifies that the antibody can capture its target in a complex mixture. The top candidate, J-IRF5-5, was then used in IP-MS on plasma samples as described below.

### Immunoprecipitation Followed by Mass Spectrometry (IP-MS) of IRF5 in Plasma

Heparin-plasma from a myositis patient and two SLE patients recruited at Karolinska University Hospital were analyzed by IP-MS as previously described (28) with a few adjustments. In brief, to an aliquot of 100 μl plasma, 400 μl of lysis buffer (1 mM Tris-HCl, 42 mM NaCl, and 0.01% NP-40 in water, pH 7.9) containing protease inhibitor cocktail (Roche) was added. Recombinant IRF5 protein (0.7 μg) was added to plasma and used as a positive control. An aliquot of 4 μg J-IRF5-5 was added to plasma samples and incubated overnight at 4°C. As negative controls a scFv antibody, generated in the same way as J-IRF5-5 but targeting an unrelated antigen, was added to plasma and to another vial, J-IRF5-5 was added to a sample without plasma (lysis buffer only). Forty microliters of anti-FLAG M2 magnetic beads was added and incubated 2–5 h at 4°C. The beads were washed three times (5–10 min in 4°C) with low salt buffer (1 mM Tris-HCl, 10 mM NaCl, and 0.01% NP-40 in water, pH 7.9) and two times with low salt buffer without NP-40. Elution was performed by 2 × 100 μl 0.5 M ammonium hydroxide and evaporated in Speedvac.

Samples were reconstituted in 50 mM ammonium bicarbonate and subsequently reduced by 1 μl of 100 mM TCEP-HCl at 37°C for 1 h, alkylated by 1 μl of 500 mM iodoacetamide in dark for 45 min and digested using 1 μg trypsin at 37°C. Sample clean-up was performed in 50 mM ammonium bicarbonate using HiPPR™ Detergent Removal Spin Column Kit according to manufacturer's instructions. Obtained peptide samples were desalted using ZipTip® pipette tips or Pierce C18 Tips (Thermo Scientific) prior mass spectrometry analysis. A standard of IRF5 peptides were generated using full length IRF5 recombinant protein applying the same digestion protocol.

Peptides were separated using an Ultimate 3000 RSLCnano system. Samples were trapped on an Acclaim PepMap nanotrap column (C18, 3 μm, 100 Å, 75 μm × 20 mm), separated on an NanoEase™ M/Z HSS column (C18, 1.8 μm, 100Å, 75 μm × 250 mm), (Thermo Scientific) and analyzed on a Q Exactive Hybrid Quadrupole-Orbitrap Mass Spectrometer (Thermo Fisher Scientific, San Jose, CA, USA). Peptides were separated using a gradient of A (3% ACN, 0.1% FA) and B (95% ACN, 0.1% FA), ranging from 3 to 40% B in 50 min with a flow of 0.25 μl/min. The Q Exactive was operated in a data dependent manner utilizing targeted SIM/ddMS^2^ method with an inclusion list containing masses corresponding to four unique IRF5 peptides. The survey scan was performed at 70.000 resolution from 400 to 1,200 m/z, with a max injection time of 100 ms and target of 1 × 10^6^ ions. For generation of HCD fragmentation spectra, a max ion injection time of 250 ms and Automated Gain Control (AGC) of 3 × 10^6^ were used before fragmentation at 30% normalized collision energy.

### Detection of IRF5 Positive Microparticles in Plasma

In another set of SLE patients (*n* = 63), citrate plasma was analyzed for detection of microparticles (MPs) expressing IRF5. Characteristics of these SLE patients and details about the sample collection can be found in Supplementary Methods and Results. Healthy controls (*n* = 20) matched for age and gender to the SLE patients were also included in this study. Platelet-poor plasma were centrifuged (2,000 g for 20 min followed by 13,000 g for 2 min) and the supernatants were then incubated with polyclonal anti-IRF5-Fluorescein isothiocyanate (FITC) (Biorbyt, UK) as described in Supplementary Methods and Results. MPs were measured by flow cytometry on a Beckman Gallios instrument (Beckman Coulter, Bream CA, USA) and were defined as particles between ~0.3 μm and 0.9 μm in size.

### Detection of IRF5 in Plasma by Sandwich ELISA

Nunc immobilizer amino plates (Thermo scientific) were coated with commercial mouse anti-human IRF5, antibody targeting aa 176–240 (Antibodies-online.com, ABIN121152). A standard curve was obtained using recombinant IRF5 protein at 0.156, 0.312, 0.625, 1.25, 2.5, 5.0, and 10 ng/ml (50 μl/well). Plasma samples from 25 SLE patients and 14 controls were diluted 1:2 in 0.1% BSA/PBS before adding 50 μl per well. As a secondary antibody, rabbit anti-human IRF5 (HPA046700, i.e., the antibody used in the antibody suspension bead array) was used. Donkey anti-rabbit IgG HRP-conjugated antibody was added for detection using TMB substrate and optical density was read at 450 nm.

### Lipid Mediators and Cytokines Data Extracted From Related Projects

In a previous study, sphingolipids were measured by LC-MS/MS in a selection of patients from our SLE cohort (29). Since one of the proteins characterizing the RF-IgM/SSA/SSB subgroup was ceramide synthase 5 (CERS5), which catalyzes the formation of C_16:0_-ceramide, we utilized data from this study where C_16:0_-ceramide was quantified. Among the analyzed patients (with data available from both C_16:0_-ceramide and antibody suspension bead array data), 16 patients were found to belong to the RF-IgM/SSA/SSB subgroup, 39 to IRF5 low subgroup and 44 patients belonged to the IRF5 high subgroup.

Previously, 20 cytokines were analyzed in plasma from the entire Karolinska SLE cohort (14), and data from cytokines relevant in inflammation, i.e., TNF-α, IL-6, IL-8, Il-10, IL-16, and IP-10, were analyzed with respect to the identified molecular subgroups in this work.

Interferon α (IFN-α) was measured by ELISA in the Karolinska SLE cohort in another study (30), and data was utilized in this work to study levels of IFN-α in IRF5 high and low subgroups. Data on IFN-α was obtained for 66% of the patients in the IRF5 low subgroup and in 70% of the patients in IRF5 high subgroup. Values below limit of quantification (LOQ) was set to LOQ/2.

### Genetic Data on IRF5

The Karolinska SLE cohort had previously been genotyped using the Immunochip Illumina Infinium Assay (31, 32). Two previously reported independent *IRF5* SLE risk variants rs4728142 and rs10488631 (a proxy to rs35000415) were selected from this data for association with IRF5 protein levels in quantitative trait locus (QTL) analyses. Genotype data was available for 253 SLE patients and 280 controls.

### Statistics

For comparison between SLE and controls Mann-Whitney U-test was used. Bonferroni-corrected *p*-value at a threshold of 0.05 was used as a measure of significance unless otherwise stated. When comparing three or more groups, i.e., when comparing the three molecular subgroups, Kruskal Wallis test or Fisher's exact test (for categorical data) was used. In addition, in Table 1, Mann–Whitney *U*-test have been used for independent comparisons between molecular subgroups in favor for scientific reasoning of selected variables with cautious interpretation of *p*-values (33, 34). Spearman rank correlation was used to investigate correlations between variables. Additional details about analysis of antibody suspension bead array data, linear modeling and K-means clustering can be found in section Data Analysis of Antibody Suspension Bead Array Data. Calculations were performed using R (19), GraphPad Prism 7 and Excel 2016. IRF5 protein QTL analysis was performed on log_10_ transformed IRF5 protein levels using linear regression in R assuming an additive genetic model.

## Results

### General Biomarker Candidates of SLE

Fifty-three antibodies, targeting 50 proteins, showed significant differences between SLE patients and controls in both sample set 1 and set 2, i.e., in two separate experiments performed in parallel containing samples from different patients/controls. In the following validation experiment, the plasma samples (*n* = 695) were analyzed using 133 antibodies targeting the 50 selected proteins. Protein profiles with low correlation (Spearman's rho < 0.40) to the screening data were removed. The remaining 15 proteins, targeted by 16 antibodies (Table 2, Figure 1F), showed a median correlation to the screening data of rho = 0.78 with a minimum correlation of rho = 0.46. Antibody target sequence for all 15 proteins can be found in Supplementary Table S-6.

**Table 2:** The 15 proteins (16 antibodies) differentially expressed comparing SLE and control.

**Protein name short**	**Full protein name**	**UniProt^a^**	**Correlation screening vs. validation^b^**	**Fold change (SLE/Ctrl)**	***p*-value (SLE vs. Ctrl)^c^**
IRF5	Interferon regulatory factor 5	Q13568	0.96	0.48	4.5E-02
SLC22A2^*^	Solute carrier family 22 (organic cation transporter), member 2	O15244	0.8	0.44	4.6E-06
S100A12^*^	S100 calcium binding protein A12	P80511	0.77	0.28	3.3E-09
RASD2^*^	GTP-binding protein Rhes	Q96D21	0.86	0.26	1.7E-05
NOS3	Nitric oxide synthase 3 (endothelial)	P29474	0.93	0.26	4.1E-02
MMP1^*^	Matrix metallopeptidase 1 (or interstitial collagenase)	P03956	0.63	0.17	3.2E-06
SPDEF^*^	SAM pointed domain containing ETS transcription factor	O95238	0.87	−0.14	1.3E-02
UBAC1	UBA domain containing 1	Q9BSL1	0.71	0.13	1.4E-04
TRIM33	Tripartite motif containing 33	Q9UPN9	0.84	0.13	3.0E-03
CFI	Complement factor I	P05156	0.65	0.13	2.9E-02
APOL6	Apolipoprotein L, 6	Q9BWW8	0.84	−0.13	4.5E-02
PPAP2A^*^	Phosphatidic acid phosphatase type 2A (or Phospholipid phosphatase 1)	O14494	0.82	0.12	9.9E-03
GRAP2	GRB2-related adaptor protein 2	O75791	0.69	0.11	3.4E-03
CRISP3^*^	Cysteine-rich secretory protein 3	P54108	0.75	−0.10	5.5E-04
CRISP3^*^	Cysteine-rich secretory protein 3	P54108	0.46	−0.10	1.8E-03
C6^*^	Complement component 6	P13671	0.68	0.10	3.7E-03

Proteins are sorted based on log-fold change between SLE samples and controls. Proteins included in suggested biomarker panel are indicated by an asterisk (“*”).

a *Protein ID in UniProt (35)*.

b *The Speaman's rho correlation coefficients for screening and validation data are reported*.

c *The highest Bonferroni-corrected p-value among set 1 and set 2 comparing SLE and Controls is reported*.

**Figure 2:**
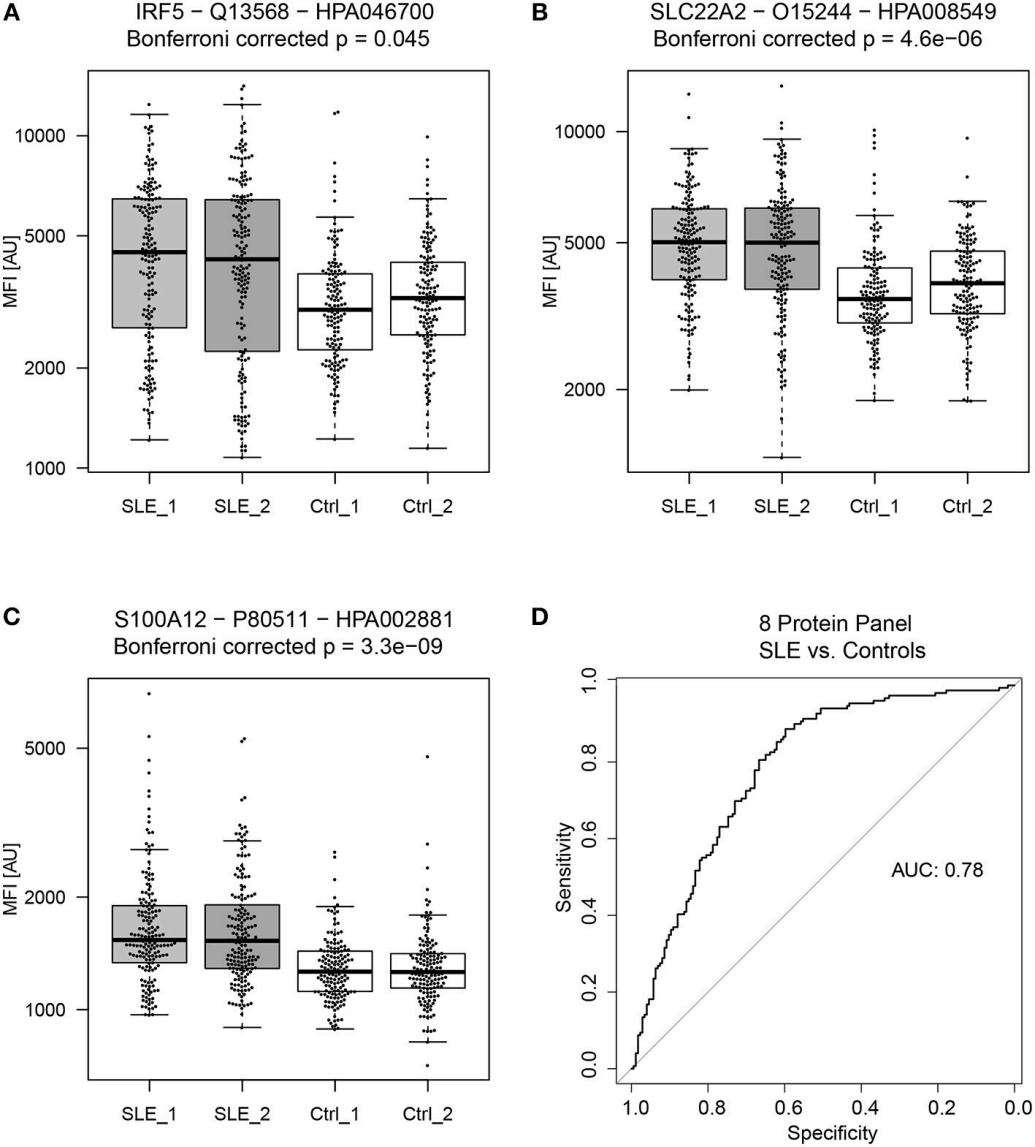
General biomarker candidates of SLE. Proteins showing the highest absolute fold change between SLE patients and controls (in both sample set 1 and 2) were **(A)** Interferon regulatory factor 5 (IRF5), **(B)** Solute carrier family 22 member 2 (SLC22A2) and **(C)** S100 calcium binding protein A12 (S100A12). A panel of 8 proteins, consisting of 9 antibodies proved to be the best panel for classifying SLE patients from controls. The panel of 8 proteins consist of RASD2, S100A12, SLC22A2, MMP1, CRISP3, C6, PPAP2A, and SPDEF and achieved an ROC AUC of 0.78 for the prediction of SLE patients and controls **(D)**.

The proteins yielding the largest fold change between SLE patients and controls (*p* < 0.05), were interferon regulatory factor 5 (IRF5), solute carrier family 22 member 2 (SLC22A2, organic cation transporter 2, OCT2) and S100 calcium binding protein A12 (S100A12, Calgranulin-C) (Figure 2). Of the 15 proteins in Table 2, three were found to be decreased, i.e., sterile alpha motif (SAM) pointed domain containing E26 transformation-specific (ETS) transcription factor (SPDEF), Apolipoprotein L6 (APOL6), and Cysteine-rich secretory protein 3 (CRISP3), and twelve were found to be increased in the SLE patients compared to controls. Seven of the proteins were classified as plasma proteins and two proteins, IRF5 and SPDEF, were transcription factors (Supplementary Table S-7). Levels of IRF5 were up-regulated and SPDEF were down-regulated in SLE compared to controls.

Proteins that showed significant differences between SLE patients and controls (Table 2), were used to create a linear model (Figure 1E). Obtained model suggested a biomarker panel of 9 antibodies, targeting 8 proteins, i.e., GTP-binding protein Rhes (RASD2), S100A12, SLC22A2, Matrix metalloproteinase-1 (MMP1), CRISP3, complement component C6 (C6), Phospholipid phosphatase 1 (PPAP2A), SPDEF, achieving a ROC AUC of 0.78 (95% CI: 0.73–0.83) for prediction of SLE patients and controls (Figure 2). In comparison, the highest achieved AUC from a single protein was 0.73 (95% CI: 0.67–0.78) for MMP1 and 0.72 (95% CI: 0.66–0.77) for S100A12, and a panel of three proteins (S100A12, SLC22A2, and PPAP2A) yielding an AUC of 0.74 (95% CI: 0.69–0.80). This panel of 8 proteins is suggested as general biomarker candidates to differentiate between SLE and controls, independently of SLE subgroups.

Applying strict statistical univariate analysis (Bonferroni correction) only two associations were identified between proteins and clinical data (i.e., serological data, clinical symptoms, disease activity scores). Lower levels of S100A12 were associated in patients with a history of lupus nephritis, i.e., “nephritis ever” defined by ACR criteria (median signal of 1591 vs. 1409, with IQR of 613 vs. 583, and Bonferroni-adjusted Mann-Whitney U-test p-value of 0.008) and IRF5 protein levels showed a weak negative correlation to C3a plasma concentration in SLE patients (Spearman's rho = −0.32, *p* < 0.0001).

### SLE Molecular Subgroups

**Figure 3:**
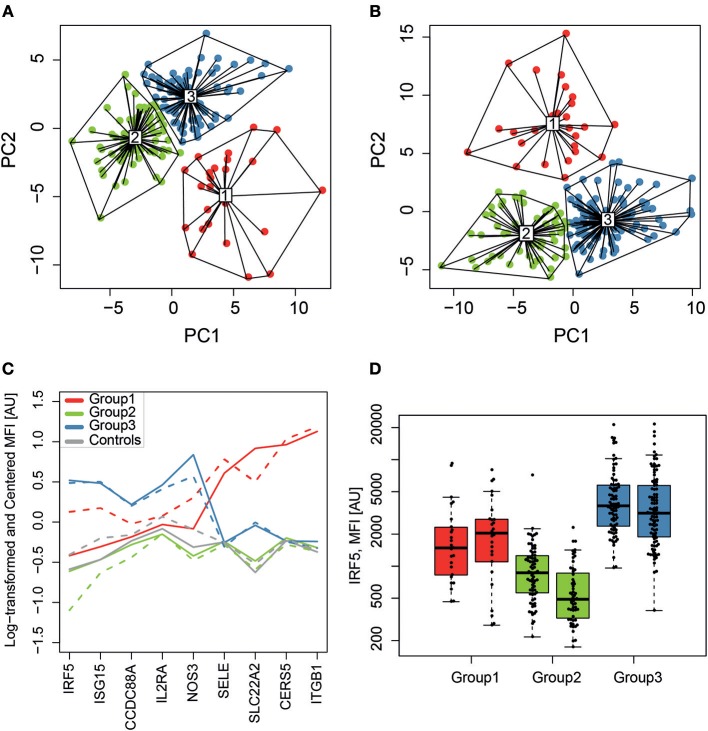
SLE molecular subgroups. K-means clustering, visualized on the two first principal components (PC1 and PC2), identified three subgroups (1-red, 2-green and 3-blue) in sample set 1 **(A)** and set 2 **(B)** with a similar clustering pattern. The relative protein profiles **(C)** of the 9 proteins with the highest loadings in both sample sets for the RF-IgM/SSA/SSB (red, *n* = 51), the IRF5 low (green, *n* = 129) and the IRF5 high (blue, *n* = 177) molecular subgroups are shown and both sample set 1 (solid line) and set 2 (dashed line) shows concordant protein profiles. It is evident that the IRF5 high subgroup discriminate from the IRF5 low subgroup based on levels of IRF5, ISG15, and NOS3, while it is evident that the RF-IgM/SSA/SSB subgroup differentiate from the other two in levels of SELE, SLC22A2, CERS5, and ITGB1. Controls are included in gray for comparison but was not included in the clustering. Levels of IRF5 **(D)** are compared between the three molecular SLE subgroups RF-IgM/SSA/SSB (red), IRF5 low (green), and IRF5 high (blue) subgroup.

Unsupervised clustering of the 281 analyzed proteins (screening data, K-means clustering) was performed to find potential molecular subgroups among the SLE patients (Figure 1D). Three distinct clusters were obtained in both experimental set 1 (Figure 3A) and set 2 (Figure 3B) and nine of the 10 proteins with the highest absolute PCA loadings were identical between the two sets. These 9 proteins were evaluated by analyzing the median protein levels and revealed concordant protein profiles between the sets (Figure 3C). This panel of biomarker candidates can be used to differentiate between suggested molecular subgroups. Molecular subgroup 1 (red, *n* = 51) showed higher levels of E-selectin (SELE), solute carrier family 22 (SLC22A2), Ceramide synthase 5 (CERS5) and Integrin subunit beta 1 (ITGB1, Glycoprotein IIA, CD29). Molecular subgroup 2 (green, *n* = 129) showed lower levels of IRF5, Ubiquitin-like protein ISG15 (ISG15), endothelial nitric oxide synthase (NOS3) and SLC22A2, and is further referred to as the IRF5 low subgroup. This subgroup was found to be similar to the control group (gray) as shown in Figure 3C. Molecular subgroup 3 (blue, *n* = 177) showed higher levels of IRF5, ISG15, NOS3, and interleukin-2 receptor subunit alpha (IL2RA) and is referred to as the IRF5 high subgroup. Levels of IRF5 in the three subgroups as well as in the two sample sets are shown in Figure 3D.

**Figure 4:**
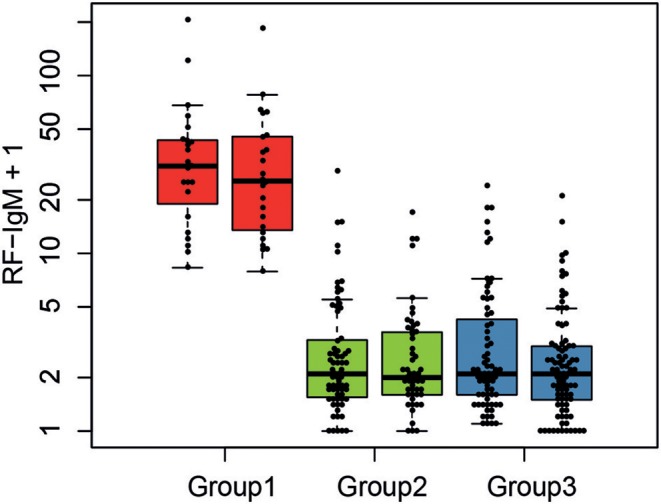
Serological characteristics of SLE molecular subgroups. The levels of RF-IgM are compared between the three molecular SLE subgroups RF-IgM/SSA/SSB (red), IRF5 low (green), and IRF5 high (blue) subgroup.

Including all available clinical and serological data, considering associations comparing all three molecular subgroups (Kruskal–Wallis test), only rheumatoid factor (RF)-IgM, RF-IgG, RF-IgA, Leptin, Fibronectin, and C3a were found to be significantly different (*p* < 0.05) in at least one subgroup after correction for multiple testing (Supplementary Figure S-2). Molecular subgroup 1 was found to have high RF-IgM levels (Figure 4) as well as high levels of autoantibodies toward Sjögren's Syndrom antigen A/B (SSA/SSB) (Supplementary Table S-8). This subgroup is further referred to as the RF-IgM/SSA/SSB subgroup. We also observed higher levels of RF-IgG and RF-IgA as well as higher levels of total IgG and IgM in this subgroup (Table 1, Supplementary Figure S-2). In addition, this subgroup showed higher frequency (45%) of patients with secondary Sjögren's syndrome (sSS), as defined according to the American-European Consensus criteria (36), compared to the IRF5 high and low subgroup (both 19%) (Supplementary Table S-9). Patients in the RF-IgM/SSA/SSB subgroup showed lower frequency of nephritis (20%) compared to IRF5 low (43%) and IRF5 high (48%) subgroups. Lower ESR were reported for the IRF5 low subgroup (Table 1). The IRF5 high subgroup was slightly older compared to other subgroups, showed lower levels of C3a and increased levels of inflammatory markers e.g., TNF-α, fibrinogen and hsCRP (Table 1).

CERS5 was, as mentioned, increased in the RF-IgM/SSA/SSB subgroup, and is an enzyme catalyzing the formation of C_16:0_-ceramide. We have previously quantified levels of sphingolipids in SLE (29) and data was available for a selection of patients. C_16:0_-ceramide levels were 415 ± 143 nM (mean ± SD) in RF-IgM/SSA/SSB subgroup (*n* = 16), 305 ± 79 nM in IRF5 low subgroup (*n* = 39) and 331 ± 84 nM in IRF5 high subgroup 3 (*n* = 44) (Supplementary Figure S-3) (Kruskal–Wallis test *p* = 0.02) supporting our finding of higher levels of CERS5 in RF-IgM/SSA/SSB subgroup.

### IRF5 in Plasma

**Figure 5:**
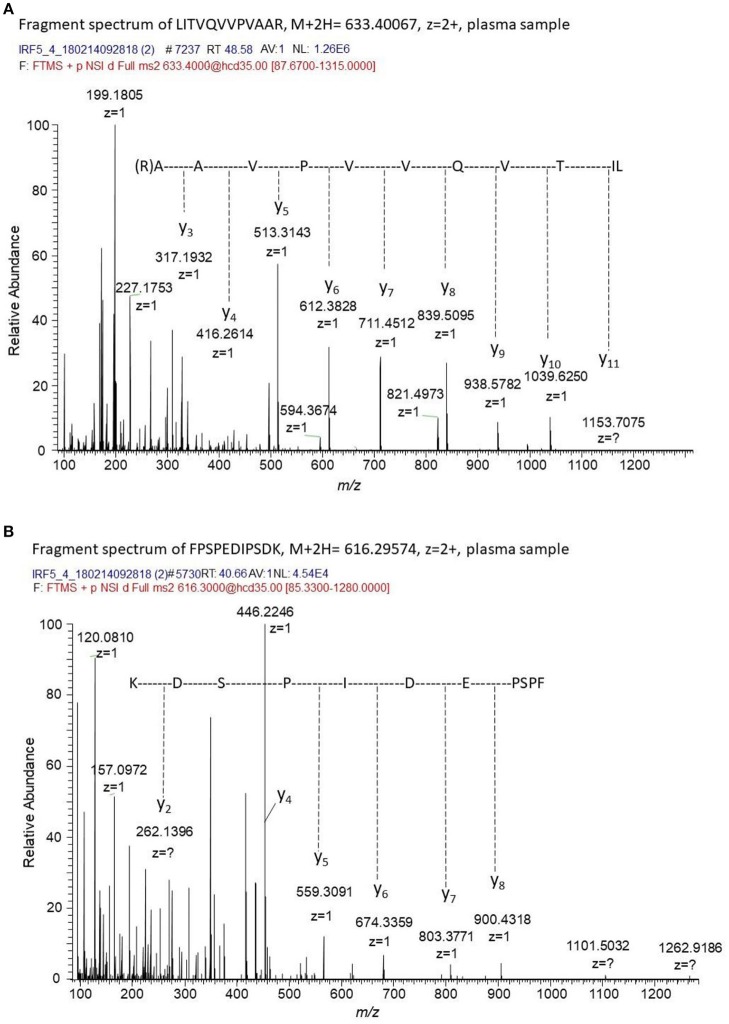
Fragment spectra of endogenous IRF5 detected in plasma. The generated recombinant antibody (J-IRF5-5) was used to capture IRF5 by immunoprecipitation in a plasma sample from a SLE patient. We obtained fragment spectra of two unique peptides of IRF5, i.e., **(A)** LITVQVVPVAAR with [M+2H]^2+^ m/z of 633.4007 eluting at a retention time of 48.6 min and **(B)** FPSPEDIPSDK with [M+2H]^2+^ m/z of 616.29574 eluting at a retention time of 40.7 min. The retention times, the masses of the unique peptides and the fragment spectra of these peptides confirms the presence of IRF5 in this plasma sample.

To confirm the presence of IRF5 in plasma immunoprecipitation tandem mass spectrometry (IP-MS/MS) was used. The targeted MS/MS method was optimized for four unique tryptic IRF5 peptides using recombinant IRF5 protein as a standard. No peaks corresponding to IRF5 were detected in the blank samples and no carry-over was observed between runs. MS/MS spectra of two of the peptides detected in plasma from a SLE patient is shown in Figure 5. IRF5 could repeatedly be detected in plasma aliquots from a myositis patient using IP-MS utilizing peptide exact mass (high-resolution m/z) and retention time. Levels were close to detection limit and fragment spectra of IRF5 peptides could not always be obtained although aliquots from the same sample were analyzed. Adding the criteria of reporting fragmentation spectra of the unique peptides, IRF5 was detected in two out of three separate experiments, not detected in one SLE patient and for the second SLE patient fragment spectra could be obtained in one out of two experiments. IP-MS, as used here, is not a quantitative method and the capture of IRF5 might slightly differ between experiments and not reach detection limit. Therefore, this is not the method of choice in a screening of the entire cohort comparing SLE and controls. Nevertheless, in cases where IRF5 was detected there is no doubt about the identity of IRF5 and that IRF5 is present in plasma. The IP, accurate retention times (RT) and high-resolution accurate-mass of unique IRF5 peptides and their fragment spectra, confirm the presence of IRF5 in the circulation.

**Figure 6:**
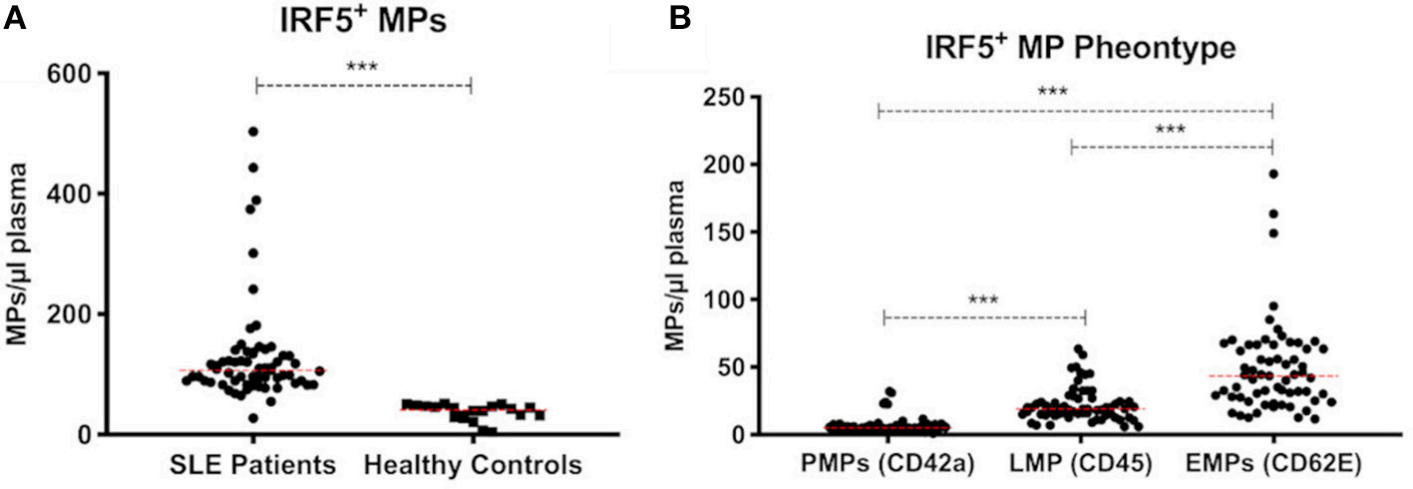
IRF5+ microparticles. Total IRF5+ MPs in SLE patients (*n* = 63) and healthy controls (*n* = 20) are shown **(A)**. IRF5+ MPs in SLE patients were phenotyped based on cell origin **(B)**. PMPs, platelet derived MPs; LMPs, leukocyte derived MPs; EMPs, Endothelial derived MPs. ^***^ < 0.001 (Mann–Whitney). Data is presented as MPs/μl plasma.

To further investigate the presence of IRF5 in plasma we analyzed IRF5 positive microparticles (MPs). The number of circulating MPs exposing IRF5 were significantly higher in SLE (*n* = 63) compared to healthy controls (*n* = 20) (130.5 ± 88 vs. 36.5 ± 14 MPs/μl, *p* < 0.0001) (Figure 6A). IRF5 positive MPs were more frequently exposed on endothelial derived MPs (CD62E+ MPs) compared to platelet and leukocyte derived MPs (*p* < 0.0001) (Figure 6B). Furthermore, total IRF5+ MPs (regardless of origin) were significantly higher in patients with higher disease activity (*p* < 0.05) (SLE activity measure (SLAM) (15) equal or above 6) (Supplementary Figure S-4).

In addition, we developed a sandwich ELISA for detection of IRF5 in plasma. IRF5 levels were significantly higher (*p* = 0.014) in SLE (*n* = 25) compared to controls (*n* = 25) (Supplementary Figure S-5). However, the sensitivity of this assay was not sufficient for screening of the entire cohort since the majority of the SLE patients analyzed (56%, *n* = 14) report levels below quantification limit. Within this data we aimed to correlate our results with the results obtained by the suspension bead array. Excluding data outside the quantitative range of the ELISA, Spearman's rank correlation analysis was performed on data from 11 SLE patients. A strong correlation (Spearman's rho = 0.63, *p* < 0.05) and a moderate R^2^ of 0.36 was obtained (Supplementary Figure S-6). However, the three samples resulting in levels above the quantification range of the ELISA, showed low or medium levels of IRF5 as measured by the suspension bead array.

*IRF5* gene polymorphism is an established risk factor in SLE (32, 37). To investigate whether IRF5 levels in plasma were regulated by known SLE genetic risk variants in *IRF5* we performed a protein quantitative trait locus analysis for two previously reported SNPs (32). We identified a weak additive association between IRF5 protein levels and the *IRF5* SLE risk variant rs4728142 (*p* = 0.003, beta = 0.07) in SLE patients and controls combined, but this effect was not apparent in either group alone. There was no association between rs10488631 and IRF5 plasma protein levels (Supplementary Figures S-7, 8).

Serum levels of IFN-α in the IRF5 high subgroup was not significantly different compared to the IRF5 low subgroup. In both subgroups 40% were defined as having detectable levels of IFN-α and the concentration (average ± SD) was 78 ± 122 pg/ml and 67 ± 149 pg/ml for the IRF5 low and IRF5 high subgroup, respectively. The number of IFN-α high patients, defined as a concentration of >100 pg/ml) was 15 in both subgroups.

## Discussion

IRF5, a transcription factor involved in regulation of interferon and cytokine production, showed the largest fold change among the differentially expressed proteins between SLE patients and controls. We also observed large variations in IRF5 levels between subgroups of SLE patients. *IRF5* gene polymorphism is a well-known risk factor in SLE (38, 39) and in several other rheumatic diseases (40). IRF5 is an intracellular protein, nevertheless we detected IRF5 in plasma using affinity-based proteomics and the extracellular location was confirmed by IP-MS in a selection of plasma samples. To further illustrate the presence of IRF5 in the circulation we report that IRF5 expressing microparticles (detected by a different antibody) are increased in SLE compared to controls.

We identified a weak positive association between IRF5 protein levels and the number of SLE risk alleles at one of two SNP representing the *IRF5* SLE genetic association. However, this effect was not apparent when separating the data from SLE patients and control individuals, thus it could be driven by the allele frequency and protein level differences between these two groups. This indicates that the *IRF5* SLE risk variants are not the sole drivers for the differences in IRF5 plasma levels that we observe. As recently discussed elsewhere (41), the contribution of IRF5 genetic risk to disease susceptibility is not known, and it is possible that *IRF5* may have both a genetic and non-genetic contribution.

It is an intriguing and novel finding that the IRF5 protein occurs in the circulation and that it stands out as a potential biomarker for SLE. The high IRF5 levels in the circulation may reflect increased cell death in SLE patients. However, the IRF5 levels also vary to a large extent within the group of SLE patients. In addition, SPDEF, another transcription factor, showed the opposite regulation in SLE plasma (10% decrease) and unless SPDEF is strongly down-regulated in SLE, the difference in IRF5 cannot solely be explained by increased cell death/loss during apoptotic clearance in patients. Reports of transcription factors in circulation are sparse (42, 43) and by our approach using antibodies designed to target a short linear sequence of the protein, it is not possible to determine if the protein is full-length or represents a splice variant or other modified product. There is no information about extracellular function of IRF5. However, the fact that IRF5 may be found on microparticles, known to mediate cell-cell signaling, merits further investigations. In addition, further studies are needed to investigate if the IRF5 protein is actively secreted and to study possible extracellular functions of IRF5.

Interestingly our unsupervised clustering of SLE patients demonstrate that IRF5 is characteristic for two different SLE subgroups. The IRF5 low subgroup also showed lower levels of ISG15, an ubiquitin-like protein that is conjugated to intracellular target proteins upon activation by IFN-α and IFN-β (44, 45), suggesting that this subgroup might be described as a less interferon dependent subgroup. On the other hand, the IRF5 high subgroup seems to be an interferon-driven subgroup with higher levels of IRF5 and ISG15 and one might speculate that patients in these two subgroups could respond differently to IFN-α-inhibition. Serum levels of IFN-α did not differ between IRF5 high and low subgroup and might be explained by that IFN-α is regulated by several genes and not only by IRF5. Building on these observations, we suggest stratification of patients based on plasma levels of IRF5 prior to clinical trials targeting the IFN pathway. These subgroups need to be further investigated, e.g., in the light of type I IFN blockers (46) not reaching primary endpoint. Stratification based on IRF5 levels may be more efficient, definitely less expensive and more suitable to implement in clinical routine, than to measure interferon signature on a gene level.

In the IRF5 high subgroup, we detected higher levels of NOS3 (endothelial (e)NOS) as compared to the IRF5 low subgroup. NOS3, an important regulator of nitric oxide (NO) production, which is essential for cardiovascular and immune functions through regulation of vascular tone, leucocyte adhesion and platelet aggregation (47, 48). NOS3 is vasoprotective and low levels of NOS3 are related to endothelial dysfunction (49). In this context, it is difficult to dissect if the low levels of NOS3 indicate an increased risk of cardiovascular events in the IRF5 low subgroup. It is also possible that the high levels of NOS3 in the IRF5 high subgroup reflect damaged blood vessels since NOS3 is expressed in the endothelium and not expected to be increased in the circulation. In the microparticles, analyzed in another set of SLE patients, the IRF5 positive microparticles were mainly of endothelial origin, suggestive of endothelial damage. CCDC88A (girdin, APE), a protein important for angiogenesis (50), was also increased in the IRF5 high subgroup and decreased in the IRF5 low subgroup. Inflammatory markers were increased in this subgroup indicating that the IRF5 high subgroup is characterized by more pronounced inflammation and one may speculate that anti-inflammatory treatment is more likely to be beneficial for this subgroup of SLE patients.

The RF-IgM/SSA/SSB subgroup is characterized by increased levels of SELE (endothelial cell adhesion molecule, E-selectin, CD62E, ICAM-1), ITGB1, SLC22A2, and CERS5. SELE is a cell adhesion glycoprotein on endothelium that can be stimulated by e.g., TNF-α (35, 51). ITGB1 is a cell surface receptor, which is part of the integrin family and it is important for cell adhesion (52). CERS5 synthesizes C16-ceramide and the increase in CERS5 was supported by the increase of C16-ceramide in this subgroup. Ceramides are signaling lipids involved in cell adhesion, inflammation as well as in a variety of other physiological functions (53, 54). This subgroup was also associated with higher levels of rheumatoid factor (RF) as well as higher levels of SSA/SSB antibodies. We previously reported high levels of RF-IgM (13), as well as higher levels of total IgG (55), in SLE patients with SSA/SSB antibodies. The RF-IgM/SSA/SSB subgroup share features with Sjögren's syndrome.

In a parallel study the same proteins were investigated but the subgroups were predefined by autoantibody profile, building on previous studies and own clinical experiences (13). The SSA/SSB+ subgroup in that study consisted of 63 patients and the largest fraction (43%) was assigned to the RF-IgM/SSA/SSB subgroup in this study, while the second largest portion (32%) was found in the IRF5 high subgroup which is in line with a pronounced interferon signaling in the SSA/SSB+ subgroup. The frequency of nephritis was similar and relatively low in both RF-IgM/SSA/SSB and SSA/SSB+ subgroups (20 and 21% respectively) while higher in other subgroups (>40%). CERS5 and ITGB1 were proteins characteristic for both RF-IgM/SSA/SSB and SSA/SSB+ subgroups. The second subgroup in our previous work, i.e., an aPL+ subgroup (*n* = 66), was to the largest extent (58%) found in the IRF5 high subgroup in this work and only 5% overlapped with the RF-IgM/SSA/SSB subgroups. Both the IRF5 high and the aPL+ subgroups were characterized by pronounced inflammation. Our conclusions are based on analysis of a large number of samples. However, validation in additional SLE cohorts and in other disease cohorts is needed.

Although validated HPA antibodies, targeting unique peptide sequences, were used, there is still a risk that these mono-specific polyclonal antibodies give rise to unspecific signals. Adding additional antibodies to the same protein enhance the probability of detecting the correct protein (56). However, the different epitopes targeted by the additional antibodies might be subject to differences in post translational modifications or differ in affinity and might not confirm the detection although the correct protein is present. In this work we confirmed the identity of one protein (IRF5) in plasma by IP-MS using a recombinant monoclonal antibody. We were also able to confirm the increased levels of IRF5 in SLE patients compared to controls in a subset of individuals utilizing a sandwich ELISA with a complementary capturing antibody. Although we did not validate the differences in IRF5 levels in subgroups of SLE in the entire cohort, we are confident of the detection of IRF5 in plasma.

Diagnostic biomarkers and novel insight into possible pathogenic pathways in SLE are of great importance and we here report a panel of biomarker candidates that could differentiate between SLE and controls. Utilizing unsupervised clustering of protein profiles, three molecular subgroups were revealed and could be characterized by another set of biomarker candidates. The RF-IgM/SSA/SSB subgroup essentially reflects the autoantibody defined SSA/SSB+ subgroup, which has previously been described (13, 55). The novel finding of circulating IRF5 protein is of importance for the other two subgroups. We suggest that stratification of patients based on circulating levels of IRF5 prior to e.g., IFN modulating treatments may be a valuable strategy. Furthermore, the IRF5 high subgroup expressed multiple signs of systemic inflammation, indicating that these patients may benefit from anti-inflammatory treatment. This work adds new information to the emerging need to classify the heterogeneous sample groups within SLE. The extension of these observations indicate that subgroups might be subject to different treatment perspectives, despite similar clinical profile.

## Ethics Statement

This study was carried out in accordance with the recommendations of The Local Ethics Committee of the Karolinska University Hospital/Karolinska Institutet in Stockholm. All subjects gave written informed consent in accordance with the Declaration of Helsinki. The protocol was approved by The Ethical Review Board in Stockholm (EPN Stockholm).

## Author Contributions

HI substantial contributions to the design of the work, data interpretation, performed IP of IRF5, and drafted the manuscript. AZ substantial contributions to the design of the work, proteomic analysis, data interpretation, and drafted the manuscript. EO performed and analyzed IP-MS experiments, and wrote sections of the manuscript. EW produced recombinant IRF5 and wrote sections of the manuscript. CP performed phage display selections of recombinant antibodies and their subsequent validation and wrote sections of the manuscript. FM analysis and interpretation of IRF5 positive MPs and wrote sections of the manuscript. AC performed analysis of lipid mediators and critically revised the manuscript. AS performed analysis of RF and critically revised the manuscript. PP and JS performed genetic data analysis and wrote sections and critically revised the manuscript. CF-C developed and supervised the ELISA analysis and critically revised the manuscript. JR interpretation of RF data and design of RF experiments and critically revised the manuscript. VO performed analysis of IFN-α and critically revised the manuscript. GG clinical evaluation of sAPS patients and critically revised the manuscript. MK clinical evaluation of sSS patients and critically revised the manuscript. AL supervision of leptin and fibronectin measurements and critically revised the manuscript. CW responsible for analysis of lipid mediators and critically revised the manuscript. AS and LR provided genetic data and critically revised the manuscript. KK responsible for MS-analysis of IRF5 and critically revised the manuscript. HP made the phage display library, supervised phage display selections and antibody validation, and wrote sections of the manuscript. SG supervision of antigen and antibody production and validation of recombinant antibodies and wrote sections of the manuscript. IG responsible for SLE cohort and clinical data and critically revised the manuscript. PN substantial contributions to the design of the work and responsible for affinity proteomic platform and drafting the manuscript. ES substantial contributions to the design of the work, responsible for SLE cohort and clinical data, and drafting the manuscript. PJ substantial contributions to the design of the work, responsible for biomarker study, and drafting the manuscript. All authors contributed to manuscript revision, read and approved the final version.

### Conflict of Interest Statement

The authors declare that the research was conducted in the absence of any commercial or financial relationships that could be construed as a potential conflict of interest.
