# Supplementary material for: Circulating Levels of Interferon Regulatory Factor-5 Associates With Subgroups of Systemic Lupus Erythematosus Patients
Source: Front Immunol. 2019 May 17;10:1029. doi: 10.3389/fimmu.2019.01029 (PMC6533644; doi:10.3389/fimmu.2019.01029)
Supplement: Supplementary file 1 [file Data_Sheet_1.docx]

**Supplementary Table S-1. Patients on treatment in different molecular SLE subgroups**

| **Medication** | **Entire SLE cohort**  **N=357** | **RF-IgM/SSA/SSB subgroup**  **N=51** | **IRF5 low subgroup**  **N=129** | **IRF5 high subgroup**  **N=177** |
| --- | --- | --- | --- | --- |
| Methotrexate | N=14 (4%) | N=3 (6%) | N=5 (4) | N=6 (3%) |
| Hydroxychloroquine | N=128 (36%) | N=22 (43%) | N=47 (36%) | N=59 (33%) |
| Azathiopine | N=60 (17%) | N=6 (12%) | N=28 (22%) | N=26 (15%) |
| Mycophenolate mofetil | N=39 (11%) | N=4 (8%) | N=12 (9%) | N=23 (13%) |
| Rituximab | N=34 (10%) | N=0 (0%) | N=16 (12%) | N=18 (10%) |
| Prednisolone dose=0 | N=147 (41%) | N=29 (57%) | N=49 (38%) | N=69 (39%) |
| Prednisolone dose<=5mg | N=244 (70%) | N=36 (71%) | N=84 (65%) | N=124 (70%) |
| Prednisolone dose>7.5mg | N=85 (24%) | N=13 (25%) | N=32 (25%) | N= 40 (23%) |

**Supplementary Table S-2** Number of samples analyzed by suspension bead arrays in total and as distributed in experimental sample set 1 and set 2. Age, gender and disease activity scores are reported.

|  | |  |  | **Number of individuals** | **Gender (F/M)** | **Age**  **mean (range)** | **SLAM (1) median (IQR)** | **SLEDAI (2) median (IQR)** |
| --- | --- | --- | --- | --- | --- | --- | --- | --- |
|  | **Entire Sample Collection** | | |  |  |  |  |  |
|  | Controls | |  | 316 | 291/25 | 46.8  (18-84) | - | - |
|  | SLE | |  | 379 | 330/49 | 46.3  (17-84) | 6 (6) | 3 (5.75) |
|  | **Set1** | |  |  |  |  |  |  |
|  | Controls | |  | 158 | 146/12 | 46.0  (18-84) | - | - |
|  | SLE | |  | 190 | 165/25 | 45.5  (17-82) | 7 (6) | 2 (6) |
|  | **Set2** | |  |  |  |  |  |  |
|  | Controls | |  | 158 | 145/13 | 47.6  (18-82) | - | - |
|  | SLE | |  | 189 | 165/24 | 47.2  (18-84) | 7 (5.75) | 3 (5) |

**Supplementary Table S-3. List of all antibodies included in the screening phase.**

| **Antibody** | **Uniprot** | **Gene** | **Gene description** |
| --- | --- | --- | --- |
| HPA000663 | P78540 | ARG2 | arginase 2 |
| HPA003545 | P18283 | GPX2 | glutathione peroxidase 2 (gastrointestinal) |
| HPA000635 | P09601 | HMOX1 | heme oxygenase (decycling) 1 |
| HPA003868 | P14174 | MIF | macrophage migration inhibitory factor (glycosylation-inhibiting factor) |
| HPA004127 | Q8TCT0 | CERK | ceramide kinase |
| HPA005788 | O75791 | GRAP2 | GRB2-related adaptor protein 2 |
| HPA005839 | Q96D21 | RASD2 | RASD family, member 2 |
| HPA003189 | P49682 | CXCR3 | chemokine (C-X-C motif) receptor 3 |
| HPA003191 | Q9BZS1 | FOXP3 | forkhead box P3 |
| HPA003748 | Q9BZS1 | FOXP3 | forkhead box P3 |
| HPA001163 | P09382 | LGALS1 | lectin, galactoside-binding, soluble, 1 |
| HPA001776 | P38646 | HSPA9 | heat shock 70kDa protein 9 (mortalin) |
| HPA005150 | P02766 | TTR | transthyretin |
| HPA000926 | P04003 | C4BPA | complement component 4 binding protein, alpha |
| HPA000931 | P42224 | STAT1 | signal transducer and activator of transcription 1, 91kDa |
| HPA000982 | P42224 | STAT1 | signal transducer and activator of transcription 1, 91kDa |
| HPA001143 | P05156 | CFI | complement factor I |
| HPA001428 | P42224 | STAT1 | signal transducer and activator of transcription 1, 91kDa |
| HPA001578 | P04003 | C4BPA | complement component 4 binding protein, alpha |
| HPA001845 | P04003 | C4BPA | complement component 4 binding protein, alpha |
| HPA002027 | P11226 | MBL2 | mannose-binding lectin (protein C) 2, soluble |
| HPA002190 | P08174 | CD55 | CD55 molecule, decay accelerating factor for complement (Cromer blood group) |
| HPA002350 | P02745 | C1QA | complement component 1, q subcomponent, A chain |
| HPA003563 | P01024 | C3 | complement component 3 |
| HPA005157 | P29597 | TYK2 | tyrosine kinase 2 |
| HPA001069 | P08575 | PTPRC | protein tyrosine phosphatase, receptor type, C |
| HPA000893 | P01011 | SERPINA3 | serpin peptidase inhibitor, clade A (alpha-1 antiproteinase, antitrypsin), member 3 |
| HPA001292 | P01009 | SERPINA1 | serpin peptidase inhibitor, clade A (alpha-1 antiproteinase, antitrypsin), member 1 |
| HPA001626 | P01009 | SERPINA1 | serpin peptidase inhibitor, clade A (alpha-1 antiproteinase, antitrypsin), member 1 |
| HPA001325 | P05231 | IL6 | interleukin 6 (interferon, beta 2) |
| HPA001410 | P01584 | IL1B | interleukin 1, beta |
| HPA001412 | P09919 | CSF3 | colony stimulating factor 3 (granulocyte) |
| HPA001554 | Q9NPF7 | IL23A | interleukin 23, alpha subunit p19 |
| HPA002274 | P11215 | ITGAM | integrin, alpha M (complement component 3 receptor 3 subunit) |
| HPA002655 | P16109 | SELP | selectin P (granule membrane protein 140kDa, antigen CD62) |
| HPA002834 | P23219 | PTGS1 | prostaglandin-endoperoxide synthase 1 (prostaglandin G/H synthase and cyclooxygenase) |
| HPA003595 | P05089 | ARG1 | arginase 1 |
| HPA004627 | P05161 | ISG15 | ISG15 ubiquitin-like modifier |
| HPA003220 | Q12901 | ZNF155 | zinc finger protein 155 |
| HPA001523 | P10809 | HSPD1 | heat shock 60kDa protein 1 (chaperonin) |
| HPA001557 | P01019 | AGT | angiotensinogen (serpin peptidase inhibitor, clade A, member 8) |
| HPA001642 | P23142 | FBLN1 | fibulin 1 |
| HPA001646 | P01042 | KNG1 | kininogen 1 |
| HPA001654 | P02749 | APOH | apolipoprotein H (beta-2-glycoprotein I) |
| HPA001888 | P02750 | LRG1 | leucine-rich alpha-2-glycoprotein 1 |
| HPA002970 | P00734 | F2 | coagulation factor II (thrombin) |
| HPA003732 | P02749 | APOH | apolipoprotein H (beta-2-glycoprotein I) |
| HPA003827 | P05160 | F13B | coagulation factor XIII, B polypeptide |
| HPA005943 | P08697 | SERPINF2 | serpin peptidase inhibitor, clade F (alpha-2 antiplasmin, pigment epithelium derived factor), member 2 |
| HPA007359 | P00734 | F2 | coagulation factor II (thrombin) |
| HPA007368 | P25774 | CTSS | cathepsin S |
| HPA003254 | Q13516 | OLIG2 | oligodendrocyte lineage transcription factor 2 |
| HPA005652 | P35548 | MSX2 | msh homeobox 2 |
| HPA002188 | P15090 | FABP4 | fatty acid binding protein 4, adipocyte |
| HPA005131 | P00797 | REN | renin |
| HPA007556 | P01344 | IGF2 | insulin-like growth factor 2 (somatomedin A) |
| HPA002267 | Q02556 | IRF8 | interferon regulatory factor 8 |
| HPA002531 | Q02556 | IRF8 | interferon regulatory factor 8 |
| HPA002881 | P80511 | S100A12 | S100 calcium binding protein A12 |
| HPA003056 | P29466 | CASP1 | caspase 1, apoptosis-related cysteine peptidase |
| HPA003620 | P80511 | S100A12 | S100 calcium binding protein A12 |
| HPA004193 | P06702 | S100A9 | S100 calcium binding protein A9 |
| HPA005938 | P24158 | PRTN3 | proteinase 3 |
| HPA003282 | P09972 | ALDOC | aldolase C, fructose-bisphosphate |
| HPA003865 | Q9UDY8 | MALT1 | mucosa associated lymphoid tissue lymphoma translocation gene 1 |
| HPA003980 | Q14116 | IL18 | interleukin 18 (interferon-gamma-inducing factor) |
| HPA004179 | P15941 | MUC1 | mucin 1, cell surface associated |
| HPA004345 | Q9UPN9 | TRIM33 | tripartite motif containing 33 |
| HPA004712 | P41235 | HNF4A | hepatocyte nuclear factor 4, alpha |
| HPA004796 | P20333 | TNFRSF1B | tumor necrosis factor receptor superfamily, member 1B |
| HPA004880 | P08582 | MFI2 | antigen p97 (melanoma associated) identified by monoclonal antibodies 133.2 and 96.5 |
| HPA004920 | P03956 | MMP1 | matrix metallopeptidase 1 (interstitial collagenase) |
| HPA007272 | P08729 | KRT7 | keratin 7 |
| HPA006189 | Q92956 | TNFRSF14 | tumor necrosis factor receptor superfamily, member 14 |
| HPA006190 | Q92956 | TNFRSF14 | tumor necrosis factor receptor superfamily, member 14 |
| HPA006404 | Q92956 | TNFRSF14 | tumor necrosis factor receptor superfamily, member 14 |
| HPA006277 | P35558 | PCK1 | phosphoenolpyruvate carboxykinase 1 (soluble) |
| HPA006225 | P16581 | SELE | selectin E |
| HPA006667 | P04062 | GBA | glucosidase, beta, acid |
| HPA006804 | P05981 | HPN | hepsin |
| HPA007009 | Q9H3D4 | TP63 | tumor protein p63 |
| HPA007040 | P50053 | KHK | ketohexokinase (fructokinase) |
| HPA007600 | O75936 | BBOX1 | butyrobetaine (gamma), 2-oxoglutarate dioxygenase (gamma-butyrobetaine hydroxylase) 1 |
| HPA007173 | Q96D42 | HAVCR1 | hepatitis A virus cellular receptor 1 |
| HPA007404 | P16471 | PRLR | prolactin receptor |
| HPA007460 | O43318 | MAP3K7 | mitogen-activated protein kinase kinase kinase 7 |
| HPA007978 | Q03431 | PTH1R | parathyroid hormone 1 receptor |
| HPA008399 | P09960 | LTA4H | leukotriene A4 hydrolase |
| HPA008130 | P03956 | MMP1 | matrix metallopeptidase 1 (interstitial collagenase) |
| HPA008262 | Q92817 | EVPL | envoplakin |
| HPA008338 | P04035 | HMGCR | 3-hydroxy-3-methylglutaryl-CoA reductase |
| HPA008424 | P14625 | HSP90B1 | heat shock protein 90kDa beta (Grp94), member 1 |
| HPA008719 | P00749 | PLAU | plasminogen activator, urokinase |
| HPA008549 | O15244 | SLC22A2 | solute carrier family 22 (organic cation transporter), member 2 |
| HPA008845 | Q16875 | PFKFB3 | 6-phosphofructo-2-kinase/fructose-2,6-biphosphatase 3 |
| HPA009083 | Q9UMF0 | ICAM5 | intercellular adhesion molecule 5, telencephalin |
| HPA009687 | O14986 | PIP5K1B | phosphatidylinositol-4-phosphate 5-kinase, type I, beta |
| HPA009426 | P16444 | DPEP1 | dipeptidase 1 (renal) |
| HPA010689 | P43115 | PTGER3 | prostaglandin E receptor 3 (subtype EP3) |
| HPA010552 | P13501 | CCL5 | chemokine (C-C motif) ligand 5 |
| HPA010534 | P49190 | PTH2R | parathyroid hormone 2 receptor |
| HPA011325 | P01127 | PDGFB | platelet-derived growth factor beta polypeptide |
| HPA011972 | P01127 | PDGFB | platelet-derived growth factor beta polypeptide |
| HPA012673 | P37088 | SCNN1A | sodium channel, non-voltage-gated 1 alpha subunit |
| HPA012511 | P02462 | COL4A1 | collagen, type IV, alpha 1 |
| HPA012770 | P01034 | CST3 | cystatin C |
| HPA012922 | Q5VYY1 | ANKRD22 | ankyrin repeat domain 22 |
| HPA013143 | P01034 | CST3 | cystatin C |
| HPA012867 | P41595 | HTR2B | 5-hydroxytryptamine (serotonin) receptor 2B, G protein-coupled |
| HPA013774 | P25101 | EDNRA | endothelin receptor type A |
| HPA014353 | Q8WWT9 | SLC13A3 | solute carrier family 13 (sodium-dependent dicarboxylate transporter), member 3 |
| HPA014404 | O76070 | SNCG | synuclein, gamma (breast cancer-specific protein 1) |
| HPA014263 | P51911 | CNN1 | calponin 1, basic, smooth muscle |
| HPA014497 | P21730 | C5AR1 | complement component 5a receptor 1 |
| HPA014606 | P48048 | KCNJ1 | potassium inwardly-rectifying channel, subfamily J, member 1 |
| HPA014405 | Q16880 | UGT8 | UDP glycosyltransferase 8 |
| HPA015014 | O43781 | DYRK3 | dual-specificity tyrosine-(Y)-phosphorylation regulated kinase 3 |
| HPA015323 | Q13627 | DYRK1A | dual-specificity tyrosine-(Y)-phosphorylation regulated kinase 1A |
| HPA015608 | Q9Y371 | SH3GLB1 | SH3-domain GRB2-like endophilin B1 |
| HPA015567 | Q9P0Z9 | PIPOX | pipecolic acid oxidase |
| HPA015715 | Q15762 | CD226 | CD226 molecule |
| HPA016613 | P04085 | PDGFA | platelet-derived growth factor alpha polypeptide |
| HPA017017 | P09960 | LTA4H | leukotriene A4 hydrolase |
| HPA014545 | P51788 | CLCN2 | chloride channel, voltage-sensitive 2 |
| HPA014862 | Q8IVM8 | SLC22A9 | solute carrier family 22 (organic anion transporter), member 9 |
| HPA017313 | P49771 | FLT3LG | fms-related tyrosine kinase 3 ligand |
| HPA017362 | Q96J88 | EPSTI1 | epithelial stromal interaction 1 (breast) |
| HPA018040 | Q9Y5I7 | CLDN16 | claudin 16 |
| HPA018148 | O95363 | FARS2 | phenylalanyl-tRNA synthetase 2, mitochondrial |
| HPA017936 | P54577 | YARS | tyrosyl-tRNA synthetase |
| HPA018526 | A6NH21 | SERINC4 | serine incorporator 4 |
| HPA018852 | P09871 | C1S | complement component 1, s subcomponent |
| HPA018120 | Q8NBS3 | SLC4A11 | solute carrier family 4, sodium borate transporter, member 11 |
| HPA017896 | P41250 | GARS | glycyl-tRNA synthetase |
| HPA019119 | P19438 | TNFRSF1A | tumor necrosis factor receptor superfamily, member 1A |
| HPA018803 | P17661 | DES | desmin |
| HPA018950 | P54577 | YARS | tyrosyl-tRNA synthetase |
| HPA018954 | P54577 | YARS | tyrosyl-tRNA synthetase |
| HPA018995 | O94907 | DKK1 | dickkopf WNT signaling pathway inhibitor 1 |
| HPA019097 | P41250 | GARS | glycyl-tRNA synthetase |
| HPA019157 | P15814 | IGLL1 | immunoglobulin lambda-like polypeptide 1 |
| HPA019543 | Q9H9B4 | SFXN1 | sideroflexin 1 |
| HPA019817 | P56748 | CLDN8 | claudin 8 |
| HPA019518 | Q14520 | HABP2 | hyaluronan binding protein 2 |
| HPA019475 | Q4G0A6 | FAM188B | family with sequence similarity 188, member B |
| HPA019502 | P25815 | S100P | S100 calcium binding protein P |
| HPA018467 | Q14005 | IL16 | interleukin 16 |
| HPA021074 | Q14520 | HABP2 | hyaluronan binding protein 2 |
| HPA021221 | P22894 | MMP8 | matrix metallopeptidase 8 (neutrophil collagenase) |
| HPA021810 | Q9HAU4 | SMURF2 | SMAD specific E3 ubiquitin protein ligase 2 |
| HPA022935 | P22894 | MMP8 | matrix metallopeptidase 8 (neutrophil collagenase) |
| HPA023099 | P05937 | CALB1 | calbindin 1, 28kDa |
| HPA023626 | P18564 | ITGB6 | integrin, beta 6 |
| HPA021125 | O95470 | SGPL1 | sphingosine-1-phosphate lyase 1 |
| HPA023599 | Q9HCG1 | ZNF160 | zinc finger protein 160 |
| HPA023684 | Q9GZX6 | IL22 | interleukin 22 |
| HPA023808 | P07358 | C8B | complement component 8, beta polypeptide |
| HPA023682 | P51451 | BLK | B lymphoid tyrosine kinase |
| HPA023680 | Q01740 | FMO1 | flavin containing monooxygenase 1 |
| HPA024099 | Q9Y2T3 | GDA | guanine deaminase |
| HPA024006 | P05089 | ARG1 | arginase 1 |
| HPA024554 | P19012 | KRT15 | keratin 15 |
| HPA026537 | Q53G44 | IFI44L | interferon-induced protein 44-like |
| HPA026589 | Q8N5B7 | CERS5 | ceramide synthase 5 |
| HPA024527 | P05121 | SERPINE1 | serpin peptidase inhibitor, clade E (nexin, plasminogen activator inhibitor type 1), member 1 |
| HPA025813 | P13010 | XRCC5 | X-ray repair complementing defective repair in Chinese hamster cells 5 (double-strand-break rejoining) |
| HPA026642 | Q9UQV4 | LAMP3 | lysosomal-associated membrane protein 3 |
| HPA027148 | Q8TCB0 | IFI44 | interferon-induced protein 44 |
| HPA027013 | O00300 | TNFRSF11B | tumor necrosis factor receptor superfamily, member 11b |
| HPA027230 | Q92630 | DYRK2 | dual-specificity tyrosine-(Y)-phosphorylation regulated kinase 2 |
| HPA026849 | Q99731 | CCL19 | chemokine (C-C motif) ligand 19 |
| HPA024087 | P49411 | TUFM | Tu translation elongation factor, mitochondrial |
| HPA028287 | P16581 | SELE | selectin E |
| HPA029874 | P49913 | CAMP | cathelicidin antimicrobial peptide |
| HPA029858 | P02778 | CXCL10 | chemokine (C-X-C motif) ligand 10 |
| HPA029165 | Q9BWW8 | APOL6 | apolipoprotein L, 6 |
| HPA029577 | P02748 | C9 | complement component 9 |
| HPA029229 | P48551 | IFNAR2 | interferon (alpha, beta and omega) receptor 2 |
| HPA029034 | Q9Y603 | ETV7 | ets variant 7 |
| HPA029167 | Q9BWW8 | APOL6 | apolipoprotein L, 6 |
| HPA029853 | O00622 | CYR61 | cysteine-rich, angiogenic inducer, 61 |
| HPA029937 | P18146 | EGR1 | early growth response 1 |
| HPA030491 | O00214 | LGALS8 | lectin, galactoside-binding, soluble, 8 |
| HPA031074 | P29279 | CTGF | connective tissue growth factor |
| HPA031456 | P03956 | MMP1 | matrix metallopeptidase 1 (interstitial collagenase) |
| HPA029035 | Q9Y603 | ETV7 | ets variant 7 |
| HPA031566 | P25942 | CD40 | CD40 molecule, TNF receptor superfamily member 5 |
| HPA030643 | P01583 | IL1A | interleukin 1, alpha |
| HPA029002 | Q9H165 | BCL11A | B-cell CLL/lymphoma 11A (zinc finger protein) |
| HPA031568 | P25942 | CD40 | CD40 molecule, TNF receptor superfamily member 5 |
| HPA028875 | P40305 | IFI27 | interferon, alpha-inducible protein 27 |
| HPA031567 | P25942 | CD40 | CD40 molecule, TNF receptor superfamily member 5 |
| HPA030546 | Q9Y275 | TNFSF13B | tumor necrosis factor (ligand) superfamily, member 13b |
| HPA029316 | P17735 | TAT | tyrosine aminotransferase |
| HPA034564 | P63165 | SUMO1 | small ubiquitin-like modifier 1 |
| HPA032146 | Q02447 | SP3 | Sp3 transcription factor |
| HPA035330 | P08758 | ANXA5 | annexin A5 |
| HPA035371 | P21549 | AGXT | alanine-glyoxylate aminotransferase |
| HPA036026 | Q8TBG4 | ETNPPL | ethanolamine-phosphate phospho-lyase |
| HPA035737 | Q16552 | IL17A | interleukin 17A |
| HPA035624 | P13501 | CCL5 | chemokine (C-C motif) ligand 5 |
| HPA035619 | P78380 | OLR1 | oxidized low density lipoprotein (lectin-like) receptor 1 |
| HPA036027 | Q8TBG4 | ETNPPL | ethanolamine-phosphate phospho-lyase |
| HPA030917 | P20591 | MX1 | myxovirus (influenza virus) resistance 1, interferon-inducible protein p78 (mouse) |
| HPA035456 | Q68CP9 | ARID2 | AT rich interactive domain 2 (ARID, RFX-like) |
| HPA030918 | P20591 | MX1 | myxovirus (influenza virus) resistance 1, interferon-inducible protein p78 (mouse) |
| HPA035370 | P21549 | AGXT | alanine-glyoxylate aminotransferase |
| HPA035769 | Q13201 | MMRN1 | multimerin 1 |
| HPA036302 | P06213 | INSR | insulin receptor |
| HPA036539 | P12081 | HARS | histidyl-tRNA synthetase |
| HPA035620 | P78380 | OLR1 | oxidized low density lipoprotein (lectin-like) receptor 1 |
| HPA037542 | Q8N0X2 | SPAG16 | sperm associated antigen 16 |
| HPA037899 | Q86VE9 | SERINC5 | serine incorporator 5 |
| HPA038013 | Q6NUJ1 | PSAPL1 | prosaposin-like 1 (gene/pseudogene) |
| HPA037958 | Q13325 | IFIT5 | interferon-induced protein with tetratricopeptide repeats 5 |
| HPA038086 | P35228 | NOS2 | nitric oxide synthase 2, inducible |
| HPA038285 | Q93088 | BHMT | betaine--homocysteine S-methyltransferase |
| HPA038785 | O14514 | BAI1 | brain-specific angiogenesis inhibitor 1 |
| HPA038845 | P11021 | HSPA5 | heat shock 70kDa protein 5 (glucose-regulated protein, 78kDa) |
| HPA038794 | P01574 | IFNB1 | interferon, beta 1, fibroblast |
| HPA040212 | P29474 | NOS3 | nitric oxide synthase 3 (endothelial cell) |
| HPA040520 | P05090 | APOD | apolipoprotein D |
| HPA040870 | P49588 | AARS | alanyl-tRNA synthetase |
| HPA041528 | P80404 | ABAT | 4-aminobutyrate aminotransferase |
| HPA040943 | Q9C0B9 | ZCCHC2 | zinc finger, CCHC domain containing 2 |
| HPA041871 | Q9HD89 | RETN | resistin |
| HPA042049 | P19827 | ITIH1 | inter-alpha-trypsin inhibitor heavy chain 1 |
| HPA041372 | Q9Y6K5 | OAS3 | 2'-5'-oligoadenylate synthetase 3, 100kDa |
| HPA041690 | P80404 | ABAT | 4-aminobutyrate aminotransferase |
| HPA040876 | Q92583 | CCL17 | chemokine (C-C motif) ligand 17 |
| HPA042608 | Q9Y5P4 | COL4A3BP | collagen, type IV, alpha 3 (Goodpasture antigen) binding protein |
| HPA043282 | P54108 | CRISP3 | cysteine-rich secretory protein 3 |
| HPA043464 | Q9Y314 | NOSIP | nitric oxide synthase interacting protein |
| HPA043691 | O15382 | BCAT2 | branched chain amino-acid transaminase 2, mitochondrial |
| HPA044402 | Q96LT4 | SAMD8 | sterile alpha motif domain containing 8 |
| HPA043956 | P20807 | CAPN3 | calpain 3, (p94) |
| HPA042924 | Q9Y617 | PSAT1 | phosphoserine aminotransferase 1 |
| HPA043823 | P13671 | C6 | complement component 6 |
| HPA045064 | O14684 | PTGES | prostaglandin E synthase |
| HPA045191 | Q86VZ5 | SGMS1 | sphingomyelin synthase 1 |
| HPA045203 | Q96DT0 | LGALS12 | lectin, galactoside-binding, soluble, 12 |
| HPA045159 | Q00987 | MDM2 | MDM2 oncogene, E3 ubiquitin protein ligase |
| HPA044768 | Q9UMW8 | USP18 | ubiquitin specific peptidase 18 |
| HPA045409 | P05121 | SERPINE1 | serpin peptidase inhibitor, clade E (nexin, plasminogen activator inhibitor type 1), member 1 |
| HPA045886 | Q16552 | IL17A | interleukin 17A |
| HPA045060 | P41597 | CCR2 | chemokine (C-C motif) receptor 2 |
| HPA045943 | Q9BZS1 | FOXP3 | forkhead box P3 |
| HPA045942 | P49682 | CXCR3 | chemokine (C-X-C motif) receptor 3 |
| HPA045794 | P07357 | C8A | complement component 8, alpha polypeptide |
| HPA045822 | P10145 | IL8 | interleukin 8 |
| HPA045827 | P29965 | CD40LG | CD40 ligand |
| HPA046579 | P02778 | CXCL10 | chemokine (C-X-C motif) ligand 10 |
| HPA046700 | Q13568 | IRF5 | interferon regulatory factor 5 |
| HPA046715 | P02647 | APOA1 | apolipoprotein A-I |
| HPA046738 | P01589 | IL2RA | interleukin 2 receptor, alpha |
| HPA046972 | P08833 | IGFBP1 | insulin-like growth factor binding protein 1 |
| HPA047290 | P07900 | HSP90AA1 | heat shock protein 90kDa alpha (cytosolic), class A member 1 |
| HPA047539 | P36888 | FLT3 | fms-related tyrosine kinase 3 |
| HPA047801 | O75952 | CABYR | calcium binding tyrosine-(Y)-phosphorylation regulated |
| HPA047815 | O14494 | PPAP2A | phosphatidic acid phosphatase type 2A |
| HPA047580 | Q9UHE5 | NAT8 | N-acetyltransferase 8 (GCN5-related, putative) |
| HPA047838 | P42771, Q8N726 | CDKN2A | cyclin-dependent kinase inhibitor 2A |
| HPA047899 | O95238 | SPDEF | SAM pointed domain containing ets transcription factor |
| HPA048012 | P21730 | C5AR1 | complement component 5a receptor 1 |
| HPA048058 | P04141 | CSF2 | colony stimulating factor 2 (granulocyte-macrophage) |
| HPA048723 | P56524 | HDAC4 | histone deacetylase 4 |
| HPA048739 | Q9UK55 | SERPINA10 | serpin peptidase inhibitor, clade A (alpha-1 antiproteinase, antitrypsin), member 10 |
| HPA048861 | Q14005 | IL16 | interleukin 16 |
| HPA048998 | P07858 | CTSB | cathepsin B |
| HPA049292 | P13726 | F3 | coagulation factor III (thromboplastin, tissue factor) |
| HPA049430 | Q9ULX3 | NOB1 | NIN1/RPN12 binding protein 1 homolog (S. cerevisiae) |
| HPA049409 | Q8WXG1 | RSAD2 | radical S-adenosyl methionine domain containing 2 |
| HPA049689 | Q9Y603 | ETV7 | ets variant 7 |
| HPA049724 | P20591 | MX1 | myxovirus (influenza virus) resistance 1, interferon-inducible protein p78 (mouse) |
| HPA049826 | Q9HA82 | CERS4 | ceramide synthase 4 |
| HPA050025 | P10809 | HSPD1 | heat shock 60kDa protein 1 (chaperonin) |
| HPA050028 | Q9BSL1 | UBAC1 | UBA domain containing 1 |
| HPA050098 | Q9C0B9 | ZCCHC2 | zinc finger, CCHC domain containing 2 |
| HPA050269 | P04196 | HRG | histidine-rich glycoprotein |
| HPA050348 | Q15762 | CD226 | CD226 molecule |
| HPA050747 | Q9NQT5 | EXOSC3 | exosome component 3 |
| HPA050798 | P78380 | OLR1 | oxidized low density lipoprotein (lectin-like) receptor 1 |
| HPA051006 | O95445 | APOM | apolipoprotein M |
| HPA051182 | P22301 | IL10 | interleukin 10 |
| HPA051370 | P02671 | FGA | fibrinogen alpha chain |
| HPA051326 | P05556 | ITGB1 | integrin, beta 1 (fibronectin receptor, beta polypeptide, antigen CD29 includes MDF2, MSK12) |
| HPA051467 | Q9UQV4 | LAMP3 | lysosomal-associated membrane protein 3 |
| HPA051514 | Q8TD30 | GPT2 | glutamic pyruvate transaminase (alanine aminotransferase) 2 |
| HPA051476 | P00734 | F2 | coagulation factor II (thrombin) |
| HPA051527 | P17735 | TAT | tyrosine aminotransferase |
| HPA051588 | Q9Y617 | PSAT1 | phosphoserine aminotransferase 1 |
| HPA052139 | P05160 | F13B | coagulation factor XIII, B polypeptide |
| HPA052256 | Q68CP9 | ARID2 | AT rich interactive domain 2 (ARID, RFX-like) |
| HPA052258 | Q16552 | IL17A | interleukin 17A |
| HPA052343 | P09238 | MMP10 | matrix metallopeptidase 10 (stromelysin 2) |
| HPA052613 | O43927 | CXCL13 | chemokine (C-X-C motif) ligand 13 |
| HPA052900 | P07602 | PSAP | prosaposin |
| HPA053140 | Q9GZX6 | IL22 | interleukin 22 |
| HPA053149 | Q9BX95 | SGPP1 | sphingosine-1-phosphate phosphatase 1 |
| HPA053247 | Q53G44 | IFI44L | interferon-induced protein 44-like |
| HPA053253 | Q9H2A7 | CXCL16 | chemokine (C-X-C motif) ligand 16 |
| HPA053294 | P02743 | APCS | amyloid P component, serum |
| HPA053433 | P09238 | MMP10 | matrix metallopeptidase 10 (stromelysin 2) |
| HPA053457 | Q9BZZ2 | SIGLEC1 | sialic acid binding Ig-like lectin 1, sialoadhesin |
| HPA053374 | P41597 | CCR2 | chemokine (C-C motif) receptor 2 |
| HPA053425 | P17174 | GOT1 | glutamic-oxaloacetic transaminase 1, soluble |
| HPA053655 | P07358 | C8B | complement component 8, beta polypeptide |
| HPA053743 | P13501 | CCL5 | chemokine (C-C motif) ligand 5 |
| HPA053965 | Q9NUQ6 | SPATS2L | spermatogenesis associated, serine-rich 2-like |
| HPA053890 | P09914 | IFIT1 | interferon-induced protein with tetratricopeptide repeats 1 |
| HPA053530 | P01579 | IFNG | interferon, gamma |
| HPA053984 | P15692 | VEGFA | vascular endothelial growth factor A |
| HPA054091 | O15382 | BCAT2 | branched chain amino-acid transaminase 2, mitochondrial |
| HPA054235 | P51451 | BLK | B lymphoid tyrosine kinase |
| HPA054317 | P07357 | C8A | complement component 8, alpha polypeptide |
| HPA054392 | P54108 | CRISP3 | cysteine-rich secretory protein 3 |
| HPA054598 | P04196 | HRG | histidine-rich glycoprotein |
| HPA054622 | P01589 | IL2RA | interleukin 2 receptor, alpha |
| HPA054772 | P78380 | OLR1 | oxidized low density lipoprotein (lectin-like) receptor 1 |
| HPA054688 | P03956 | MMP1 | matrix metallopeptidase 1 (interstitial collagenase) |
| HPA054698 | P00734 | F2 | coagulation factor II (thrombin) |
| HPA054950 | P15924 | DSP | desmoplakin |
| HPA054954 | P02778 | CXCL10 | chemokine (C-X-C motif) ligand 10 |
| HPA055096 | P04114 | APOB | apolipoprotein B |
| HPA055046 | P16410 | CTLA4 | cytotoxic T-lymphocyte-associated protein 4 |
| HPA055048 | O00744 | WNT10B | wingless-type MMTV integration site family, member 10B |
| HPA055211 | P07476 | IVL | involucrin |
| HPA055245 | Q9HCE7 | SMURF1 | SMAD specific E3 ubiquitin protein ligase 1 |
| HPA055380 | P09914 | IFIT1 | interferon-induced protein with tetratricopeptide repeats 1 |
| HPA055427 | Q9NUQ6 | SPATS2L | spermatogenesis associated, serine-rich 2-like |
| HPA055622 | P05305 | EDN1 | endothelin 1 |
| HPA055707 | O95238 | SPDEF | SAM pointed domain containing ets transcription factor |
| HPA055752 | P10599 | TXN | thioredoxin |
| HPA055754 | P05413 | FABP3 | fatty acid binding protein 3, muscle and heart (mammary-derived growth inhibitor) |
| HPA055858 | Q9HBU1 | BARX1 | BARX homeobox 1 |
| HPA056073 | Q9NR20 | DYRK4 | dual-specificity tyrosine-(Y)-phosphorylation regulated kinase 4 |
| HPA056226 | O95445 | APOM | apolipoprotein M |
| HPA056465 | Q99502 | EYA1 | eyes absent homolog 1 (Drosophila) |
| HPA056614 | Q7L5A8 | FA2H | fatty acid 2-hydroxylase |
| HPA056956 | P63165 | SUMO1 | small ubiquitin-like modifier 1 |
| HPA057105 | P26639 | TARS | threonyl-tRNA synthetase |
| HPA057255 | Q9Y6K8 | AK5 | adenylate kinase 5 |
| HPA057322 | P41159 | LEP | leptin |
| HPA057382 | Q68CP9 | ARID2 | AT rich interactive domain 2 (ARID, RFX-like) |
| HPA057404 | P04141 | CSF2 | colony stimulating factor 2 (granulocyte-macrophage) |
| HPA055738 | Q9UII4 | HERC5 | HECT and RLD domain containing E3 ubiquitin protein ligase 5 |
| HPA057804 | Q96PD4 | IL17F | interleukin 17F |
| HPA057891 | P16581 | SELE | selectin E |
| HPA058037 | Q92583 | CCL17 | chemokine (C-C motif) ligand 17 |
| HPA058310 | Q93088 | BHMT | betaine--homocysteine S-methyltransferase |
| HPA058312 | P29475 | NOS1 | nitric oxide synthase 1 (neuronal) |
| HPA058537 | P00505 | GOT2 | glutamic-oxaloacetic transaminase 2, mitochondrial |
| HPA058095 | Q8TBG4 | ETNPPL | ethanolamine-phosphate phospho-lyase |
| HPA058204 | Q8N5Z0 | AADAT | aminoadipate aminotransferase |
| HPA057179 | P10145 | IL8 | interleukin 8 |
| HPA059138 | Q5XPI4 | RNF123 | ring finger protein 123 |
| HPA059130 | P01133 | EGF | epidermal growth factor |
| HPA060034 | Q9Y2P8 | RCL1 | RNA terminal phosphate cyclase-like 1 |
| HPA060372 | Q53G44 | IFI44L | interferon-induced protein 44-like |
| HPA060803 | P04054 | PLA2G1B | phospholipase A2, group IB (pancreas) |
| HPA061287 | Q02410 | APBA1 | amyloid beta (A4) precursor protein-binding, family A, member 1 |
| HPA061393 | Q96RN1 | SLC26A8 | solute carrier family 26, member 8 |
| HPA061320 | Q96J88 | EPSTI1 | epithelial stromal interaction 1 (breast) |
| HPA061464 | P05164 | MPO | myeloperoxidase |
| HPA061408 | Q3V6T2 | CCDC88A | coiled-coil domain containing 88A |
| HPA061825 | P12081 | HARS | histidyl-tRNA synthetase |
| HPA062132 | Q9Y314 | NOSIP | nitric oxide synthase interacting protein |

**Supplementary Table S-4. List of all antibodies included in the validation phase.**

| **Antibody** | **Uniprot** | **Gene** | **Gene description** |
| --- | --- | --- | --- |
| HPA000663 | P78540 | ARG2 | arginase 2 |
| HPA000806 | Q9BWW8 | APOL6 | apolipoprotein L, 6 |
| HPA005788 | O75791 | GRAP2 | GRB2-related adaptor protein 2 |
| HPA005839 | Q96D21 | RASD2 | RASD family, member 2 |
| HPA003189 | P49682 | CXCR3 | chemokine (C-X-C motif) receptor 3 |
| HPA001668 | Q9BSL1 | UBAC1 | UBA domain containing 1 |
| HPA005651 | Q9BSL1 | UBAC1 | UBA domain containing 1 |
| HPA005762 | Q9BSL1 | UBAC1 | UBA domain containing 1 |
| HPA001539 | P05121 | SERPINE1 | serpin peptidase inhibitor, clade E (nexin, plasminogen activator inhibitor type 1), member 1 |
| HPA001554 | Q9NPF7 | IL23A | interleukin 23, alpha subunit p19 |
| HPA002834 | P23219 | PTGS1 | prostaglandin-endoperoxide synthase 1 (prostaglandin G/H synthase and cyclooxygenase) |
| HPA001523 | P10809 | HSPD1 | heat shock 60kDa protein 1 (chaperonin) |
| HPA002881 | P80511 | S100A12 | S100 calcium binding protein A12 |
| HPA003620 | P80511 | S100A12 | S100 calcium binding protein A12 |
| HPA005938 | P24158 | PRTN3 | proteinase 3 |
| HPA003036 | O00622 | CYR61 | cysteine-rich, angiogenic inducer, 61 |
| HPA003901 | P14625 | HSP90B1 | heat shock protein 90kDa beta (Grp94), member 1 |
| HPA004345 | Q9UPN9 | TRIM33 | tripartite motif containing 33 |
| HPA004426 | P07602 | PSAP | prosaposin |
| HPA004920 | P03956 | MMP1 | matrix metallopeptidase 1 (interstitial collagenase) |
| HPA006189 | Q92956 | TNFRSF14 | tumor necrosis factor receptor superfamily, member 14 |
| HPA006190 | Q92956 | TNFRSF14 | tumor necrosis factor receptor superfamily, member 14 |
| HPA006404 | Q92956 | TNFRSF14 | tumor necrosis factor receptor superfamily, member 14 |
| HPA006405 | Q92956 | TNFRSF14 | tumor necrosis factor receptor superfamily, member 14 |
| HPA006225 | P16581 | SELE | selectin E |
| HPA006288 | Q9H3D4 | TP63 | tumor protein p63 |
| HPA006289 | Q9H3D4 | TP63 | tumor protein p63 |
| HPA007009 | Q9H3D4 | TP63 | tumor protein p63 |
| HPA007010 | Q9H3D4 | TP63 | tumor protein p63 |
| HPA007713 | P03956 | MMP1 | matrix metallopeptidase 1 (interstitial collagenase) |
| HPA007146 | Q96D42 | HAVCR1 | hepatitis A virus cellular receptor 1 |
| HPA007173 | Q96D42 | HAVCR1 | hepatitis A virus cellular receptor 1 |
| HPA008130 | P03956 | MMP1 | matrix metallopeptidase 1 (interstitial collagenase) |
| HPA008719 | P00749 | PLAU | plasminogen activator, urokinase |
| HPA008738 | P00749 | PLAU | plasminogen activator, urokinase |
| HPA008567 | O15244 | SLC22A2 | solute carrier family 22 (organic cation transporter), member 2 |
| HPA008549 | O15244 | SLC22A2 | solute carrier family 22 (organic cation transporter), member 2 |
| HPA012939 | P37088 | SCNN1A | sodium channel, non-voltage-gated 1 alpha subunit |
| HPA013773 | P25101 | EDNRA | endothelin receptor type A |
| HPA013774 | P25101 | EDNRA | endothelin receptor type A |
| HPA014087 | P25101 | EDNRA | endothelin receptor type A |
| HPA014315 | P51911 | CNN1 | calponin 1, basic, smooth muscle |
| HPA014633 | O76070 | SNCG | synuclein, gamma (breast cancer-specific protein 1) |
| HPA012743 | P37088 | SCNN1A | sodium channel, non-voltage-gated 1 alpha subunit |
| HPA017896 | P41250 | GARS | glycyl-tRNA synthetase |
| HPA019097 | P41250 | GARS | glycyl-tRNA synthetase |
| HPA019956 | Q14005 | IL16 | interleukin 16 |
| HPA018467 | Q14005 | IL16 | interleukin 16 |
| HPA021124 | Q96DT0 | LGALS12 | lectin, galactoside-binding, soluble, 12 |
| HPA021159 | Q14005 | IL16 | interleukin 16 |
| HPA024061 | P05156 | CFI | complement factor I |
| HPA025295 | P16581 | SELE | selectin E |
| HPA026537 | Q53G44 | IFI44L | interferon-induced protein 44-like |
| HPA026597 | Q96DT0 | LGALS12 | lectin, galactoside-binding, soluble, 12 |
| HPA026781 | P03956 | MMP1 | matrix metallopeptidase 1 (interstitial collagenase) |
| HPA025713 | P16581 | SELE | selectin E |
| HPA027230 | Q92630 | DYRK2 | dual-specificity tyrosine-(Y)-phosphorylation regulated kinase 2 |
| HPA027227 | P16581 | SELE | selectin E |
| HPA028381 | O00622 | CYR61 | cysteine-rich, angiogenic inducer, 61 |
| HPA028066 | O95238 | SPDEF | SAM pointed domain containing ETS transcription factor |
| HPA028287 | P16581 | SELE | selectin E |
| HPA028168 | O00622 | CYR61 | cysteine-rich, angiogenic inducer, 61 |
| HPA029165 | Q9BWW8 | APOL6 | apolipoprotein L, 6 |
| HPA029577 | P02748 | C9 | complement component 9 |
| HPA029229 | P48551 | IFNAR2 | interferon (alpha, beta and omega) receptor 2 |
| HPA029167 | Q9BWW8 | APOL6 | apolipoprotein L, 6 |
| HPA029853 | O00622 | CYR61 | cysteine-rich, angiogenic inducer, 61 |
| HPA029937 | P18146 | EGR1 | early growth response 1 |
| HPA031456 | P03956 | MMP1 | matrix metallopeptidase 1 (interstitial collagenase) |
| HPA029938 | P18146 | EGR1 | early growth response 1 |
| HPA029316 | P17735 | TAT | tyrosine aminotransferase |
| HPA035646 | Q9Y5P4 | COL4A3BP | collagen, type IV, alpha 3 (Goodpasture antigen) binding protein |
| HPA036026 | Q8TBG4 | ETNPPL | ethanolamine-phosphate phospho-lyase |
| HPA035645 | Q9Y5P4 | COL4A3BP | collagen, type IV, alpha 3 (Goodpasture antigen) binding protein |
| HPA036027 | Q8TBG4 | ETNPPL | ethanolamine-phosphate phospho-lyase |
| HPA035457 | Q68CP9 | ARID2 | AT rich interactive domain 2 (ARID, RFX-like) |
| HPA037958 | Q13325 | IFIT5 | interferon-induced protein with tetratricopeptide repeats 5 |
| HPA038102 | Q3V6T2 | CCDC88A | coiled-coil domain containing 88A |
| HPA038101 | Q3V6T2 | CCDC88A | coiled-coil domain containing 88A |
| HPA037957 | Q13325 | IFIT5 | interferon-induced protein with tetratricopeptide repeats 5 |
| HPA040212 | P29474 | NOS3 | nitric oxide synthase 3 (endothelial cell) |
| HPA040870 | P49588 | AARS | alanyl-tRNA synthetase |
| HPA039288 | P04141 | CSF2 | colony stimulating factor 2 (granulocyte-macrophage) |
| HPA040943 | Q9C0B9 | ZCCHC2 | zinc finger, CCHC domain containing 2 |
| HPA040711 | Q9C0B9 | ZCCHC2 | zinc finger, CCHC domain containing 2 |
| HPA042608 | Q9Y5P4 | COL4A3BP | collagen, type IV, alpha 3 (Goodpasture antigen) binding protein |
| HPA043282 | P54108 | CRISP3 | cysteine-rich secretory protein 3 |
| HPA044223 | P49588 | AARS | alanyl-tRNA synthetase |
| HPA044459 | P16581 | SELE | selectin E |
| HPA043823 | P13671 | C6 | complement component 6 |
| HPA043187 | Q68CP9 | ARID2 | AT rich interactive domain 2 (ARID, RFX-like) |
| HPA045203 | Q96DT0 | LGALS12 | lectin, galactoside-binding, soluble, 12 |
| HPA044546 | Q8TBG4 | ETNPPL | ethanolamine-phosphate phospho-lyase |
| HPA045886 | Q16552 | IL17A | interleukin 17A |
| HPA045942 | P49682 | CXCR3 | chemokine (C-X-C motif) receptor 3 |
| HPA045058 | P14625 | HSP90B1 | heat shock protein 90kDa beta (Grp94), member 1 |
| HPA046738 | P01589 | IL2RA | interleukin 2 receptor, alpha |
| HPA046972 | P08833 | IGFBP1 | insulin-like growth factor binding protein 1 |
| HPA047815 | O14494 | PPAP2A | phosphatidic acid phosphatase type 2A |
| HPA047822 | P04054 | PLA2G1B | phospholipase A2, group IB (pancreas) |
| HPA047899 | O95238 | SPDEF | SAM pointed domain containing ETS transcription factor |
| HPA048058 | P04141 | CSF2 | colony stimulating factor 2 (granulocyte-macrophage) |
| HPA048546 | P01583 | IL1A | interleukin 1, alpha |
| HPA048861 | Q14005 | IL16 | interleukin 16 |
| HPA049169 | P02743 | APCS | amyloid P component, serum |
| HPA049792 | Q9BZZ2 | SIGLEC1 | sialic acid binding Ig-like lectin 1, sialoadhesin |
| HPA049856 | P14625 | HSP90B1 | heat shock protein 90kDa beta (Grp94), member 1 |
| HPA050014 | P14625 | HSP90B1 | heat shock protein 90kDa beta (Grp94), member 1 |
| HPA050025 | P10809 | HSPD1 | heat shock 60kDa protein 1 (chaperonin) |
| HPA050028 | Q9BSL1 | UBAC1 | UBA domain containing 1 |
| HPA050039 | P05121 | SERPINE1 | serpin peptidase inhibitor, clade E (nexin, plasminogen activator inhibitor type 1), member 1 |
| HPA050098 | Q9C0B9 | ZCCHC2 | zinc finger, CCHC domain containing 2 |
| HPA050640 | P08833 | IGFBP1 | insulin-like growth factor binding protein 1 |
| HPA051502 | P03956 | MMP1 | matrix metallopeptidase 1 (interstitial collagenase) |
| HPA051527 | P17735 | TAT | tyrosine aminotransferase |
| HPA052256 | Q68CP9 | ARID2 | AT rich interactive domain 2 (ARID, RFX-like) |
| HPA052258 | Q16552 | IL17A | interleukin 17A |
| HPA052343 | P09238 | MMP10 | matrix metallopeptidase 10 (stromelysin 2) |
| HPA052750 | O14494 | PPAP2A | phosphatidic acid phosphatase type 2A |
| HPA052900 | P07602 | PSAP | prosaposin |
| HPA053247 | Q53G44 | IFI44L | interferon-induced protein 44-like |
| HPA053433 | P09238 | MMP10 | matrix metallopeptidase 10 (stromelysin 2) |
| HPA053457 | Q9BZZ2 | SIGLEC1 | sialic acid binding Ig-like lectin 1, sialoadhesin |
| HPA053372 | P13671 | C6 | complement component 6 |
| HPA054392 | P54108 | CRISP3 | cysteine-rich secretory protein 3 |
| HPA054622 | P01589 | IL2RA | interleukin 2 receptor, alpha |
| HPA054688 | P03956 | MMP1 | matrix metallopeptidase 1 (interstitial collagenase) |
| HPA055424 | P56748 | CLDN8 | claudin 8 |
| HPA055707 | O95238 | SPDEF | SAM pointed domain containing ETS transcription factor |
| HPA056902 | Q92630 | DYRK2 | dual-specificity tyrosine-(Y)-phosphorylation regulated kinase 2 |
| HPA057382 | Q68CP9 | ARID2 | AT rich interactive domain 2 (ARID, RFX-like) |
| HPA057404 | P04141 | CSF2 | colony stimulating factor 2 (granulocyte-macrophage) |
| HPA057891 | P16581 | SELE | selectin E |
| HPA058467 | P48551 | IFNAR2 | interferon (alpha, beta and omega) receptor 2 |
| HPA058613 | O00300 | TNFRSF11B | tumor necrosis factor receptor superfamily, member 11b |
| HPA058095 | Q8TBG4 | ETNPPL | ethanolamine-phosphate phospho-lyase |
| HPA060337 | P29474 | NOS3 | nitric oxide synthase 3 (endothelial cell) |
| HPA060539 | Q9BZZ2 | SIGLEC1 | sialic acid binding Ig-like lectin 1, sialoadhesin |
| HPA060803 | P04054 | PLA2G1B | phospholipase A2, group IB (pancreas) |
| HPA060605 | P56748 | CLDN8 | claudin 8 |
| HPA061408 | Q3V6T2 | CCDC88A | coiled-coil domain containing 88A |
| HPA062006 | P04141 | CSF2 | colony stimulating factor 2 (granulocyte-macrophage) |
| HPA062180 | Q13325 | IFIT5 | interferon-induced protein with tetratricopeptide repeats 5 |
| HPA063008 | P18146 | EGR1 | early growth response 1 |
| HPA063044 | Q68CP9 | ARID2 | AT rich interactive domain 2 (ARID, RFX-like) |
| HPA064018 | Q96D42 | HAVCR1 | hepatitis A virus cellular receptor 1 |
| HPA064787 | O75791 | GRAP2 | GRB2-related adaptor protein 2 |
| HPA065850 | P16581 | SELE | selectin E |
| HPA066344 | P56748 | CLDN8 | claudin 8 |
| HPA066215 | Q68CP9 | ARID2 | AT rich interactive domain 2 (ARID, RFX-like) |
| HPA066763 | P03956 | MMP1 | matrix metallopeptidase 1 (interstitial collagenase) |
| HPA067301 | P16581 | SELE | selectin E |
| HPA067300 | Q68CP9 | ARID2 | AT rich interactive domain 2 (ARID, RFX-like) |
| HPA067382 | Q96DT0 | LGALS12 | lectin, galactoside-binding, soluble, 12 |
| HPA068433 | P29474 | NOS3 | nitric oxide synthase 3 (endothelial cell) |
| HPA068770 | O95238 | SPDEF | SAM pointed domain containing ETS transcription factor |
| HPA069135 | P09238 | MMP10 | matrix metallopeptidase 10 (stromelysin 2) |
| HPA001143 | P05156 | CFI | complement factor I |
| HPA002190 | P08174 | CD55 | CD55 molecule, decay accelerating factor for complement (Cromer blood group) |
| HPA001069 | P08575 | PTPRC | protein tyrosine phosphatase, receptor type, C |
| HPA004627 | P05161 | ISG15 | ISG15 ubiquitin-like modifier |
| HPA008424 | P14625 | HSP90B1 | heat shock protein 90kDa beta (Grp94), member 1 |
| HPA012673 | P37088 | SCNN1A | sodium channel, non-voltage-gated 1 alpha subunit |
| HPA014404 | O76070 | SNCG | synuclein, gamma (breast cancer-specific protein 1) |
| HPA014263 | P51911 | CNN1 | calponin 1, basic, smooth muscle |
| HPA019119 | P19438 | TNFRSF1A | tumor necrosis factor receptor superfamily, member 1A |
| HPA018803 | P17661 | DES | desmin |
| HPA019817 | P56748 | CLDN8 | claudin 8 |
| HPA024527 | P05121 | SERPINE1 | serpin peptidase inhibitor, clade E (nexin, plasminogen activator inhibitor type 1), member 1 |
| HPA030643 | P01583 | IL1A | interleukin 1, alpha |
| HPA035737 | Q16552 | IL17A | interleukin 17A |
| HPA035456 | Q68CP9 | ARID2 | AT rich interactive domain 2 (ARID, RFX-like) |
| HPA045409 | P05121 | SERPINE1 | serpin peptidase inhibitor, clade E (nexin, plasminogen activator inhibitor type 1), member 1 |
| HPA046700 | Q13568 | IRF5 | interferon regulatory factor 5 |
| HPA053294 | P02743 | APCS | amyloid P component, serum |
| HPA060372 | Q53G44 | IFI44L | interferon-induced protein 44-like |

**Supplementary Table S-5.** IP-MS results using J-IRF5-5 in HEK293 cells after addition of recombinant IRF5 protein. Top 20 proteins are sorted by decreasing spectral counts.

| **Accession^a^** | **Description** | **NSAF^b^** |
| --- | --- | --- |
| Q13568 | Interferon regulatory factor 5 OS=Homo sapiens GN=**IRF5** PE=1 SV=2 - [IRF5_HUMAN] | 107 |
| O43707 | Alpha-actinin-4 OS=Homo sapiens GN=ACTN4 PE=1 SV=2 - [ACTN4_HUMAN] | 82 |
| P62805 | Histone H4 OS=Homo sapiens GN=HIST1H4A PE=1 SV=2 - [H4_HUMAN] | 75 |
| P04264 | Keratin, type II cytoskeletal 1 OS=Homo sapiens GN=KRT1 PE=1 SV=6 - [K2C1_HUMAN] | 59 |
| P08107 | Heat shock 70 kDa protein 1A/1B OS=Homo sapiens GN=HSPA1A PE=1 SV=5 - [HSP71_HUMAN] | 53 |
| P35527 | Keratin, type I cytoskeletal 9 OS=Homo sapiens GN=KRT9 PE=1 SV=3 - [K1C9_HUMAN] | 46 |
| P98175 | RNA-binding protein 10 OS=Homo sapiens GN=RBM10 PE=1 SV=3 - [RBM10_HUMAN] | 45 |
| Q07955 | Serine/arginine-rich splicing factor 1 OS=Homo sapiens GN=SRSF1 PE=1 SV=2 - [SRSF1_HUMAN] | 43 |
| O75688 | Protein phosphatase 1B OS=Homo sapiens GN=PPM1B PE=1 SV=1 - [PPM1B_HUMAN] | 40 |
| P13645 | Keratin, type I cytoskeletal 10 OS=Homo sapiens GN=KRT10 PE=1 SV=6 - [K1C10_HUMAN] | 39 |
| P35908 | Keratin, type II cytoskeletal 2 epidermal OS=Homo sapiens GN=KRT2 PE=1 SV=2 - [K22E_HUMAN] | 36 |
| P34931 | Heat shock 70 kDa protein 1-like OS=Homo sapiens GN=HSPA1L PE=1 SV=2 - [HS71L_HUMAN] | 35 |
| P09651 | Heterogeneous nuclear ribonucleoprotein A1 OS=Homo sapiens GN=HNRNPA1 PE=1 SV=5 - [ROA1_HUMAN] | 34 |
| P07437 | Tubulin beta chain OS=Homo sapiens GN=TUBB PE=1 SV=2 - [TBB5_HUMAN] | 33 |
| P68371 | Tubulin beta-4B chain OS=Homo sapiens GN=TUBB4B PE=1 SV=1 - [TBB4B_HUMAN] | 33 |
| P62701 | 40S ribosomal protein S4, X isoform OS=Homo sapiens GN=RPS4X PE=1 SV=2 - [RS4X_HUMAN] | 30 |
| O14744 | Protein arginine N-methyltransferase 5 OS=Homo sapiens GN=PRMT5 PE=1 SV=4 - [ANM5_HUMAN] | 28 |
| P16403 | Histone H1.2 OS=Homo sapiens GN=HIST1H1C PE=1 SV=2 - [H12_HUMAN] | 28 |
| P23588 | Eukaryotic translation initiation factor 4B OS=Homo sapiens GN=EIF4B PE=1 SV=2 - [IF4B_HUMAN] | 26 |
| Q6P3W7 | SCY1-like protein 2 OS=Homo sapiens GN=SCYL2 PE=1 SV=1 - [SCYL2_HUMAN] | 24 |

a Uniprot ID (1)

b Normalized spectral abundance factor (NSAF) (2)

1. The UniProt Consortium. UniProt: the universal protein knowledgebase. Nucleic Acids Research. 2017;45(D1):D158-D69.

2. Florens L, Carozza MJ, Swanson SK, Fournier M, Coleman MK, Workman JL, Washburn MP. Analyzing chromatin remodeling complexes using shotgun proteomics and normalized spectral abundance factors. Methods (San Diego, Calif). 2006;40(4):303-11.

**Supplementary Table S-6.** Antibody target sequence of the 16 antibodies that showed significance between SLE and controls, *i.e.*, proteins shown in Table 2.

| **Protein Name short** | **Antibody ID in HPA (3)^a^** | **Protein Epitope Signature Tag** |
| --- | --- | --- |
|  |  |  |
| IRF5 | HPA046700 | NKSRDFRLIYDGPRDMPPQPYKIYEVCSNGPAPTDSQPPEDYSFGAGEEEE |
| SLC22A2 | HPA008549 | ESPRWLISQNKNAEAMRIIKHIAKKNGKSLPASLQRLRLEEETGKKLNPSFLDLVRTPQIR |
| S100A12 | HPA002881 | KLEEHLEGIVNIFHQYSVRKGHFDTLSKGELKQLLTKELANTIKNIKDKAVIDEIFQGLDANQDEQVDFQEFISLVAIALKAAHYHTHKE |
| RASD2 | HPA005839 | DTSGNHPFPAMRRLSILTGDVFILVFSLDNRESFDEVKRLQKQILEVKSCLKNKTKEAAELPMVICGNKNDHGELCRQVPTTEAELLVSGDENCAYFEVSAKKNTNVDEMFYVLFSMAKLPHEMSPALHRKISVQYGDAFHPRPFCMRRVKEMDAYGMVSPFARRPSVNSDLKYIKAKVLR |
| NOS3 | HPA040212 | ENGESFAAALMEMSGPYNSSPRPEQHKSYKIRFNSISCSDPLVSSWRRKRKESSNTDSAGALGTLRFCVFGLGS |
| MMP1 | HPA031456 | PATLETQEQDVDLVQKYLEKYYNLKNDGRQVEKRRNSGPVVEKLKQMQEFFGLKVTGKPDAETLKVMKQPRCGVPDVAQFVLTEGNPRWEQTHLTYRIENYTPDLPRADVDHAIEKAFQLWSNVTPLTFTKVSEGQADIMISFVRGDH |
| SPDEF | HPA047899 | QCPVIDSQAPAGSLDLVPGGLTLEEHSLEQVQSMVVGEVLKDIETACKLLNITADPMDWSPSNVQKW |
| UBAC1 | HPA050028 | QEEKIFAGKVLRLHICASDGAEWLEEATEDTSVEKLKERCLKHCAHGSLEDPKSITHHKLIHAASERVLSDARTILEENIQDQDVLLLIKKRAPSPLPKMADVSAEEKKKQDQKAPDKEAILRATANLPSYNMDRAAVQTNMRD |
| TRIM33 | HPA004345 | MQPHLQRQHSNPGHAGPFPVVSVHNTTINPTSPTTATMANANRGPTSPSVTAIELIPSVTNPENLPSLPDIPPIQLEDAGSSSLDNLLSRYISGSHLPPQPTSTMNPSPGPSALSPGSSGLSNSHTPVRPPSTSSTGSR |
| CFI | HPA001143 | GCWILTAAHCLRASKTHRYQIWTTVVDWIHPDLKRIVIEYVDRIIFHENYNAGTYQNDIALIEMKKDGNKKDCELPRSIPACVPWSPYLFQPNDTCIVSGWGREKDNERVFSLQWGEVKLISNCSKFYGNRFYEKEMECAGTYDG |
| APOL6 | HPA029165 | GTLERSKNKEAQARAEDILPTYDQEDREDEEEK |
| PPAP2A | HPA047815 | DFFKERTSFKERKEEDSHTTLHETPTTGNHYPSNHQP |
| GRAP2 | HPA005788 | TNSISRQKQIFLRDRTREDQGHRGNSLDRRSQGGPHLSGAVGEEIRPSMNRKLSDHPPTLPLQQHQHQPQPPQYAPAPQQLQQPPQQRYLQHHHFHQERRGGSLDINDGHCGTGLGSEMNAALMHRRHTDPVQLQAAGRVRWARALYDFE |
| CRISP3 | HPA054392 | SCPDNCDDGLCTNGCKYEDLYSNCKSLKLTLTCKHQLVRDSCKASCNCSNS |
| CRISP3 | HPA043282 | SCPDNCDDGLCTNGCKYEDLYSNCKSLKLTLTCKHQLVRDSCKASCNCSNS |
| C6 | HPA043823 | GLTEEEAKHCVRIETKKRVLFAKKTKVEHRCTTNKLSEKHEGSFIQGAEKSISLIRGGRSEYGAALAWEKGSSGLEEKTFSEWLESVKENPAVIDFELA |

**^a^ Isoforms targeted by each antibody are reported in the Human Protein Atlas (HPA,** [**www.proteinatlas.org**](http://www.proteinatlas.org)**). For antibody targeting IRF5 (HPA046700), the following splice variants can be captured: IRF5-001 - ENSP00000385352 [100%], IRF5-002 - ENSP00000349770 [100%], IRF5-003 - ENSP00000418037 [100%], IRF5-005 - ENSP00000419950 [100%], IRF5-009 - ENSP00000419149 [100%], IRF5-201 - ENSP00000249375 [100%], IRF5-202 - ENSP00000480058 [100%] and IRF5-008 - ENSP00000417770 [92%].**

**Supplementary Table S-7.** Classification of selected proteins into protein classes. Transcription factors and plasma proteins are highlighted in bold.

| **Protein name short** | **Protein name** | **Protein class** (4) |
| --- | --- | --- |
| IRF5 | interferon regulatory factor 5 | Disease related genes, Predicted intracellular proteins, **Transcription factors** |
| SLC22A2 | solute carrier family 22 (organic cation transporter), member 2 | Predicted membrane proteins, Transporters |
| S100A12 | S100 calcium binding protein A12 | **Plasma proteins**, Predicted intracellular proteins |
| RASD2 | GTP-binding protein Rhes | Predicted intracellular proteins |
| NOS3 | nitric oxide synthase 3 (endothelial) | Cancer-related genes, Disease related genes, Enzymes, FDA approved drug targets, **Plasma proteins**, Predicted intracellular proteins |
| MMP1 | matrix metallopeptidase 1 (interstitial collagenase) | Cancer-related genes, Candidate cardiovascular disease genes, Enzymes, FDA approved drug targets, **Plasma proteins**, Predicted secreted proteins |
| SPDEF^#^ | SAM pointed domain containing ETS transcription factor | Predicted intracellular proteins, **Transcription factors** |
| UBAC1 | UBA domain containing 1 | Predicted intracellular proteins |
| TRIM33 | tripartite motif containing 33 | Predicted intracellular proteins |
| CFI | complement factor I | Disease related genes, Enzymes, **Plasma proteins**, Potential drug targets, Predicted secreted proteins |
| APOL6^#^ | apolipoprotein L, 6 | Predicted membrane proteins |
| PPAP2A | phosphatidic acid phosphatase type 2A | Enzymes, Predicted membrane proteins |
| GRAP2 | GRB2-related adaptor protein 2 | **Plasma proteins**, Predicted intracellular proteins |
| CRISP3^#^ | cysteine-rich secretory protein 3 | **Plasma proteins**, Predicted intracellular proteins, Predicted secreted proteins |
| C6 | complement component 6 | Cancer-related genes, Disease related genes, **Plasma proteins**, Potential drug targets, Predicted secreted proteins, Transporters |

^#^ Down-regulated in SLE compared to controls.

**Supplementary Table S-8. Serological, complement and cytokine data for the three molecular subgroups as well as for the entire cohort**

|  | Entire SLE cohort ^c^ | Molecular SLE subgroups ^c^ | | | Comparing SLE subgroups ^d^ | | |
| --- | --- | --- | --- | --- | --- | --- | --- |
|  | n=357 | RF-IgM/ SSA/SSB subgroup  N=51 | IRF5 low subgroup  N=129 | IRF5 high subgroup  N=177 | RF-IgM/ SSA/SSB vs. IRF5 low subgroup | RF-IgM/ SSA/SSB vs IRF5 high subgroup | IRF5 low  vs IRF5 high subgroup |
| SSA Ro52 Mx ^a^ | 0.9  (0.9-2.7) | 6.7  (0.9-9) | 0.9  (0.9-0.9) | 0.9  (0.9-0.9) | **P<0.0001** | **P<0.0001** | P=0.47 |
| SSA Ro60 Mx ^a^ | 0.9  (0.9-9) | 9  (0.9-9) | 0.9  (0.9-8.1) | 0.9  (0.9-9) | **P<0.0001** | **P<0.0001** | P=0.86 |
| SSB Mx ^b^ | 0.9  (0.9-0.9) | 5.1  (0.9-9) | 0.9  (0.9-0.9) | 0.9  (0.9-0.9) | **P<0.0001** | **P<0.0001** | P=0.96 |
| SmRNP mx ^a^ | 0.9  (0.9-0.95) | 0.9  (0.9-0.9) | 0.9  (0.9-2.85) | 0.  (0.9-0.95) | P=0.56 | P=0.86 | P=0.46 |
| Ds DNA Mx ^a^ | 5  (4-19.5) | 4  (4-10) | 5  (4-18.5) | 5  (4-23) | P=0.15 | P=0.87 | P=0.99 |
| p-albumin (g/l) ^a^ | 39 (36-42) | 39 (37-42) | 40 (36.5-42) | 38 (35-41) | P=0.42 | P=0.32 | P=0.02 |
| U-albumin/ creatinine (mg/mmol) ^a^ | 1.1 (0.54-5.5) | 0.71 (0.44-2.4) | 1.04 (0.55-5.7) | 1.48 (0.59-12.1) | P=0.06 | P=0.004 | P=0.26 |
| C4 (g/l) ^a^ | 0.15  (0.1-0.2) | 0.15  (0.11-0.19) | 0.15  (0.1-0.2) | 0.14  (0.1-0.2) | P=0.97 | P=0.83 | P=0.76 |
| C3 (g/l) ^a^ | 0.87  (0.71-1.04) | 0.92  (0.72-1.04) | 0.85  (0.71-1.04) | 0.86  (0.71-1.03) | P=0.50 | P=0.50 | P=0.82 |
| C2 (% of normal) ^a^ | 120  (94-145) | 135.5  (106-161) | 114  (87-139) | 121  (95-139.3) | P=0.007 | P=0.04 | P=0.25 |
| C3dg (mg/l) ^a^ | 7.6  (5.6-9.7) | 6.25  (4.4-8.9) | 7.4  (5.9-9.6) | 8.1  (5.6-9.7) | P=0.16 | P=0.09 | P=0.56 |
| C1q KS% ^a^ | 103  (83-119) | 107  (75-121.5) | 102  (78-115) | 103  (86-122) | P=0.40 | P=0.90 | P=0.14 |
| Leucocytes (x109/l) ^a^ | 5.1 (3.6-6.8) | 4 (3-6.1) | 5.2 (4.0-7.0) | 5.2 (3.8-7.0) | P=0.006 | P=0.003 | P=0.90 |
| Lymphocytes (x109/l) ^a^ | 1.1 (0.8-1.5) | 0.9 (0.7-1.3) | 1.2 (0.8-1.6) | 1.1 (0.8-1.5) | P=0.03 | P=0.03 | P=0.95 |
| Neutrophils (x109/l) ^a^ | 3.1 (2.2-4.8) | 2.6 (1.7-3.6) | 3.2 (2.2-5) | 3.2 (2.2-4.9) | P=0.02 | P=0.006 | P=0.97 |
| B thrombocytes ^a^ | 231.5 (187.5-287.8) | 227 (174-285) | 223 (189-286.5) | 242 (185.8-292.3) | P=0.96 | P=0.47 | P=0.31 |
| TG (mmol/l) ^a^ | 1.0 (0.68-1.48) | 0.9 (0.6-1.4) | 1.0 (0.67-1.4) | 1.0 (0.7-1.6) | P=0.83 | P=0.13 | P=0.07 |
| TNF-α (pg/ml) ^b^ | 4.5 (3.3-6.2) | 4.8 (3.5-6.7) | 4.0 (2.8-5.7) | 5.1 (3.6-6.4) | P=0.015 | P=0.77 | **P=0.0005** |
| IL-6 (pg/ml) ^b^ | 1.4 (0.8-2.3) | 1.2 (0.8-2.2) | 1.2 (0.8-2.0) | 1.5 (0.9-2.6) | P=0.93 | P=0.26 | P=0.09 |
| IL-8 (pg/ml) ^b^ | 5.1 (3.2-9.0) | 5.2 (2.8-8.7) | 4.1 (2.9-7.6) | 5.8 (3.6-10.1) | P=0.28 | P=0.41 | P=0.004 |
| Il-10 (pg/ml) ^b^ | 0.88 (0.6-1.7) | 1.1 (0.6 -1.8) | 0.86 (0.5-1.8) | 0.84 (0.6-1.4) | P=0.30 | P=0.16 | P=0.96 |
| IL-16 (pg/ml) ^b^ | 215 (154-287) | 177 (129-250) | 216 (159-284) | 225 (159-310) | P=0.02 | P=0.004 | P=0.46 |
| IP-10 (pg/ml) ^b^ | 743 (449-1369) | 966 (566-1803) | 606 (406-1150) | 781 (504-1433) | P=0.003 | P=0.12 | P=0.01 |

^a^ Serology data and complement data obtained as described in previous work. (5)

^b^ Cytokine data obtained from (7)

^c^ Median (25% quantile - 75% quantile), NR = not reported.

^d^ Mann-Whitney U-test for pairwise comparison of subgroups was used to characterize subgroups. P-values <0.001 without adjustment for multiple testing are highlighted in bold.

**Supplementary Table S-9. Frequency data on sSS and sAPS in the three molecular subgroups as well as in respect to autoantibodies related to APS.**

|  | Entire SLE cohort n=357 | RF-IgM/ SSA/SSB subgroup  N=51 | IRF5 low subgroup  N=129 | IRF5 high subgroup  N=177 |
| --- | --- | --- | --- | --- |
| sSS (8) | N=81  23% | N=23  45% | N=25  19% | N=33  19% |
| sAPS (9) | N=33  9% | N=2  4% | N=8  6% | N=23  13% |
| B2GP1 IgG  Mx (Yes) | N=53 of 274  19% | N=4 of 44  2% | N=18 of 99  18% | N=31 of 131  24% |
| B2GP1 IgM  Mx (Yes) | N=25  9% | N=1  2% | N=13  13% | N=11  8% |
| B2GP1 IgA  Mx (Yes) | N=44  16% | N=2  4% | N=18  18% | N=24  18% |
| CL IgG  Mx (Yes) | N=52  19% | N=4  9% | N=19  19% | N=29  22% |
| CL IgM  Mx (Yes) | N=23  8% | N=1  2% | N=10  10% | N=13  9% |
| CL IgA  Mx (Yes) | N=46  17% | N=3  7% | N=19  19% | N=24  18% |
| LA | N=64  18% | N=5  10% | N=19  15% | N=40  23% |

LA = Lupus anticoagulans

1. Liang MH, Socher SA, Roberts WN, Esdail JM. Measurement of systemic lupus erythematosus activity in clinical research. *Arthritis Rheum* (1988) 31. doi: 10.1002/art.1780310701.

2. Gladman DD, Ibañez D, Urowitz MB. Systemic lupus erythematosus disease activity index 2000. *The Journal of rheumatology* (2002) 29(2):288.

3. Uhlen M, Hober S, Wernerus H, Björling L, Ponten F, Oksvold P, et al. Towards a knowledge-based Human Protein Atlas. *Nature biotechnology* (2010) 28(12):1248-50. doi: 10.1038/nbt1210-1248.

4. The UniProt Consortium. UniProt: the universal protein knowledgebase. *Nucleic Acids Research* (2017) 45(D1):D158-D69. doi: 10.1093/nar/gkw1099.

5. Idborg H, Zandian A, Sandberg A-S, Nilsson B, Elvin K, Truedsson L, et al. Two subgroups in systemic lupus erythematosus with features of antiphospholipid or Sjögren’s syndrome differ in molecular signatures and treatment perspectives. *Arthritis Research & Therapy* (2019) 21(1):62. doi: 10.1186/s13075-019-1836-8.

6. Idborg H, Zandian A, Sandberg A, Nilsson B, Elvin K, Truedsson L, et al. Two subgroups in Systemic Lupus Erythematosus with Features of Antiphospholipid or Sjögren's Syndrome, differ in molecular signatures and treatment perspectives. *Arthritis Research & Therapy* (2018) Accepted for publication after minor revisions. Resubmission before 25th December 2018.

7. Idborg H, Eketjäll S, Pettersson S, Gustafsson JT, Zickert A, Kvarnström M, et al. TNF-α and plasma albumin as biomarkers of disease activity in systemic lupus erythematosus. *Lupus science & medicine* (2018) 5(1):e000260. doi: 10.1136/lupus-2018-000260.

8. Vitali C, Bombardieri S, Jonsson R, Moutsopoulos HM, Alexander EL, Carsons SE, et al. Classification criteria for Sjögren's syndrome: a revised version of the European criteria proposed by the American-European Consensus Group. *Annals of the rheumatic diseases* (2002) 61(6):554.

9. Miyakis S, Lockshin MD, Atsumi T, Branch DW, Brey RL, Cervera R, et al. International consensus statement on an update of the classification criteria for definite antiphospholipid syndrome (APS). *Journal of Thrombosis and Haemostasis* (2006) 4(2):295-306. doi: 10.1111/j.1538-7836.2006.01753.x.
