# Supplementary material for: Circulating Levels of Interferon Regulatory Factor-5 Associates With Subgroups of Systemic Lupus Erythematosus Patients
Source: Front Immunol. 2019 May 17;10:1029. doi: 10.3389/fimmu.2019.01029 (PMC6533644; doi:10.3389/fimmu.2019.01029)
Supplement: Supplementary file 2 [file Data_Sheet_2.docx]

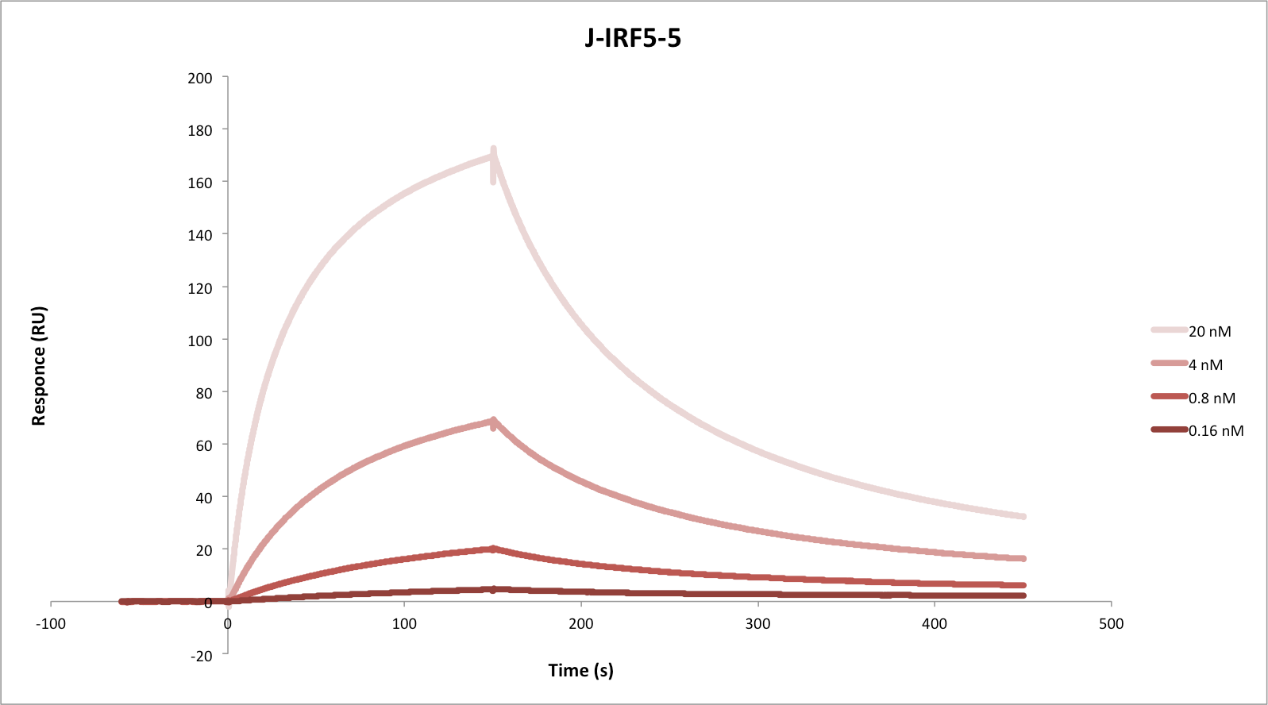


**Supplementary Figure S-1**

Multiple Cycle Kinetics (MCK) using full length IRF5: The anti-FLAG M2 antibody (Sigma-Aldrich, St. Louis, MO, USA)), functioning as a capture molecule, was covalently coupled to a Series S CM5 amine sensor chip according to the manufacturer’s instructions. FLAG-tagged scFv were caught onto the surface, and IRF5, full length as well as the E232-L434 construct, was added at a flow rate of 30 ul/min. Four concentrations of antigen (0.16, 0.8, 4, and 20 nM) were injected, in a multi-cycle kinetic mode. The anti-FLAG antibody surface was regenerated with 10 mM glycine-HCl pH 2.5. The experiment was performed at 25°C and using HBS-EP+ (GE Healthcare) as running buffer. By subtracting the signal of a reference surface, an anti-FLAG antibody-coupled surface, response curves for the scFvs were obtained. The BIAcore T200 Evaluation 3.1 software was used for analyses of the response curves and reaction rate kinetics calculated using the predefined 1:1 Langmuir binding model.

**A**

**B**

**C**

**D**

**E**

**F**

**Supplementary Figure S-2.** The three molecular SLE subgroups (RF-IgM/SSA/SSB –red, IRF5 low – green, IRF5 high –blue) showed significant differences (Kruskal-Wallis test p-value <0.05) in the following clinical variables: higher RF-IgM (A), IgG (B), IgA (C) in the RF-IgM/SSA/SSB subgroup, higher leptin levels (D), lower fibronectin levels (E) and lower C3a levels (F) in the IRF5 high subgroup.

**
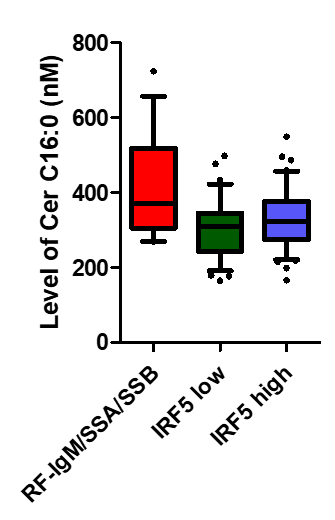
**

**Supplementary Figure S-3.** The level of C_16:0_-ceramide was measured in a selection of SLE patients in a previous study based on our SLE cohort (1). The level of C_16:0_-ceramide was increased in the RF-IgM/SSA/SSB subgroup compared to the IRF5 low subgroup (Kruskal-Wallis with Dunns post-hoc test p=0.02; Mann Whitney U-test, p=0.007).


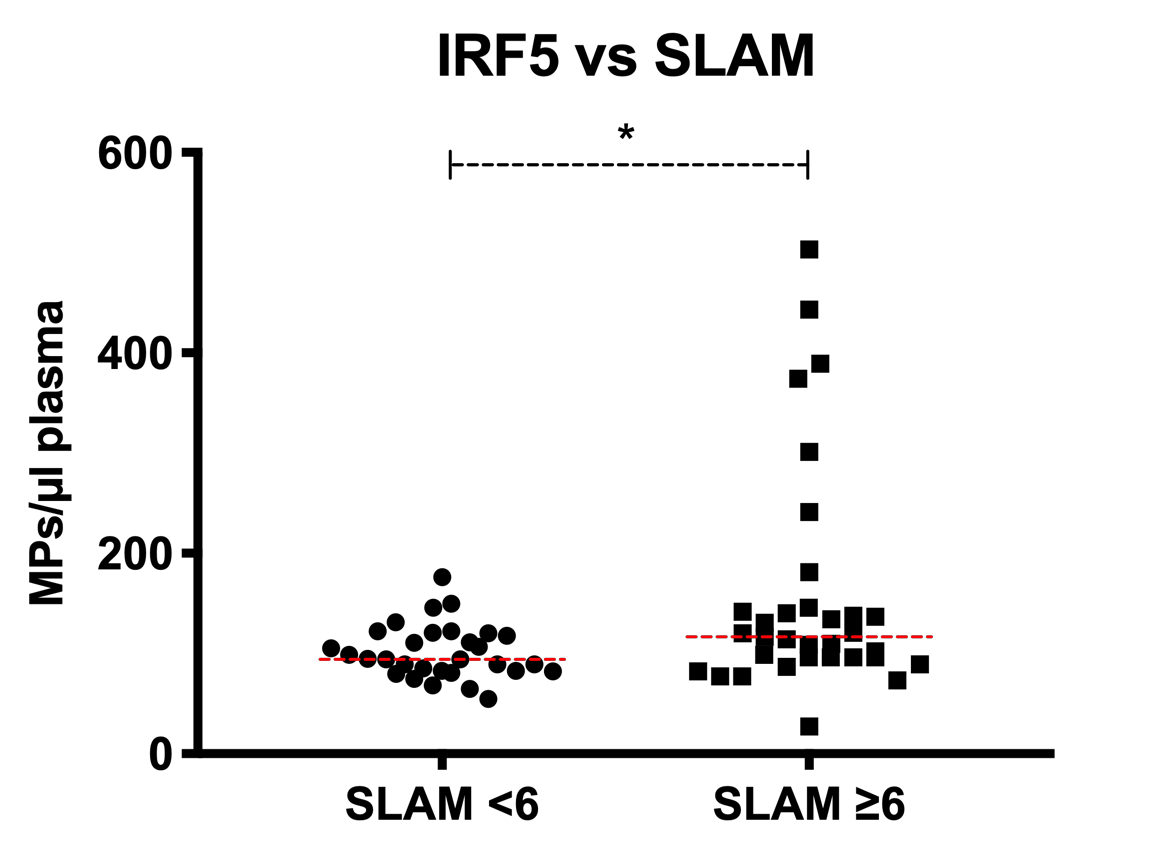


Supplementary Figure S-4. IRF5+ MPs and disease activity as measured by SLAM. * p<0.05 (Mann-Whitney)


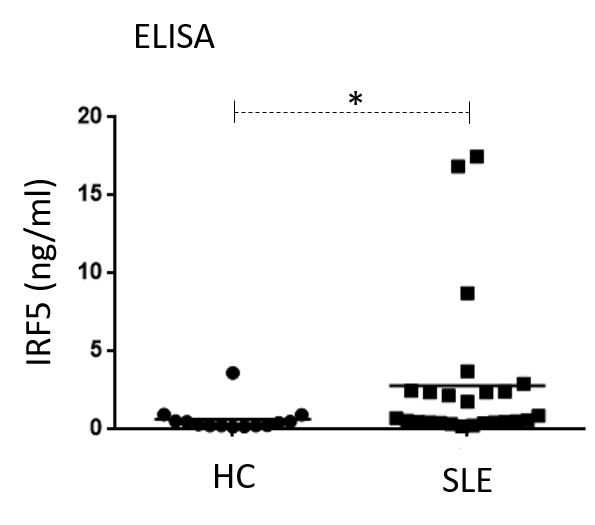


**Supplementary Figure S-5**. Levels of IRF5 in plasma were detected using sandwich ELISA in a subset of healthy controls (n=14) and SLE patients (n=25). Higher levels (p = 0.014) was detected in SLE patients. ELISA was performed by coating Nunc immobilizer amino plates (Thermo scientific) with commercial mouse anti-human IRF5, an antibody targeting aa 176-240 (Antibodies-online.com, ABIN121152). Incubation overnight was performed by adding 100 µl/well at 1 µg/ml 100 mM sodium phosphate buffer. The wells were washed three times with PBS 0.5% Tween (300 µl/well) and blocked using 1% BSA/PBS for 60 min. A standard curve was obtained using recombinant IRF5 protein at 0.156, 0.312, 0.625, 1.25, 2.5, 5.0 and 10 ng/ml (50µl/well). Plasma samples from 25 SLE patients and 14 controls were diluted 1:2 in 0.1 % BSA/PBS before adding 50µl per well. Incubation with samples was 1-2 hours in room temperature and thereafter the plate was washed three times with PBS 0.05% Tween (300µl/well). As a secondary antibody, rabbit anti-human IRF5 (HPA046700, *i.e*. the antibody used in the antibody suspension bead array) was used (1:700) and incubated 1-2 hours in room temperature. After wash, donkey anti-rabbit IgG HRP-conjugated antibody was added and incubated for 1 hour. After wash, TBM substrate was added (100µl/well) and when dark blue the reaction was stopped with 1M H_2_SO_4_ and OD was read at 450 nm.

A

B

**Supplementary Figure S-6**. Comparison of IRF5 levels measured by suspension bead array. A) All data obtained for both SLE (n=25) and controls (n=14) utilizing ELISA are shown. B) Data within the quantitative range of the ELISA, *i.e*. 0.5-8 ng/ml are shown for SLE patients (n=11. A correlation of Spearman’s rho = 0.63 (p<0.05) and a R^2^ of 0.36 were obtained for ELISA and suspension bead array data.


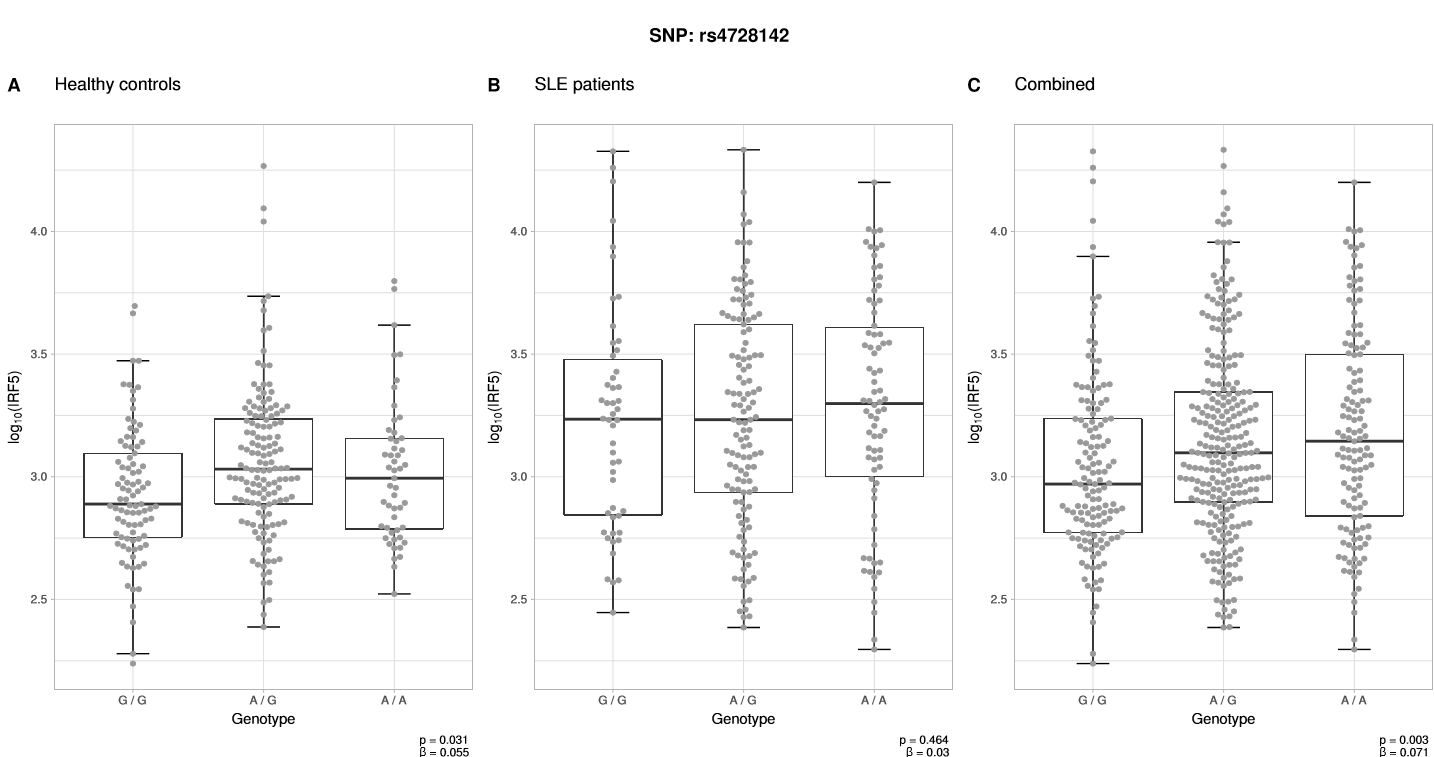


**Supplementary Figure S-7**. Associations between log_10_ IRF5 protein levels and the *IRF5* SLE risk variant rs4728142 are shown for A) healthy controls, B) SLE patients and C) both SLE patients and healthy controls combined.


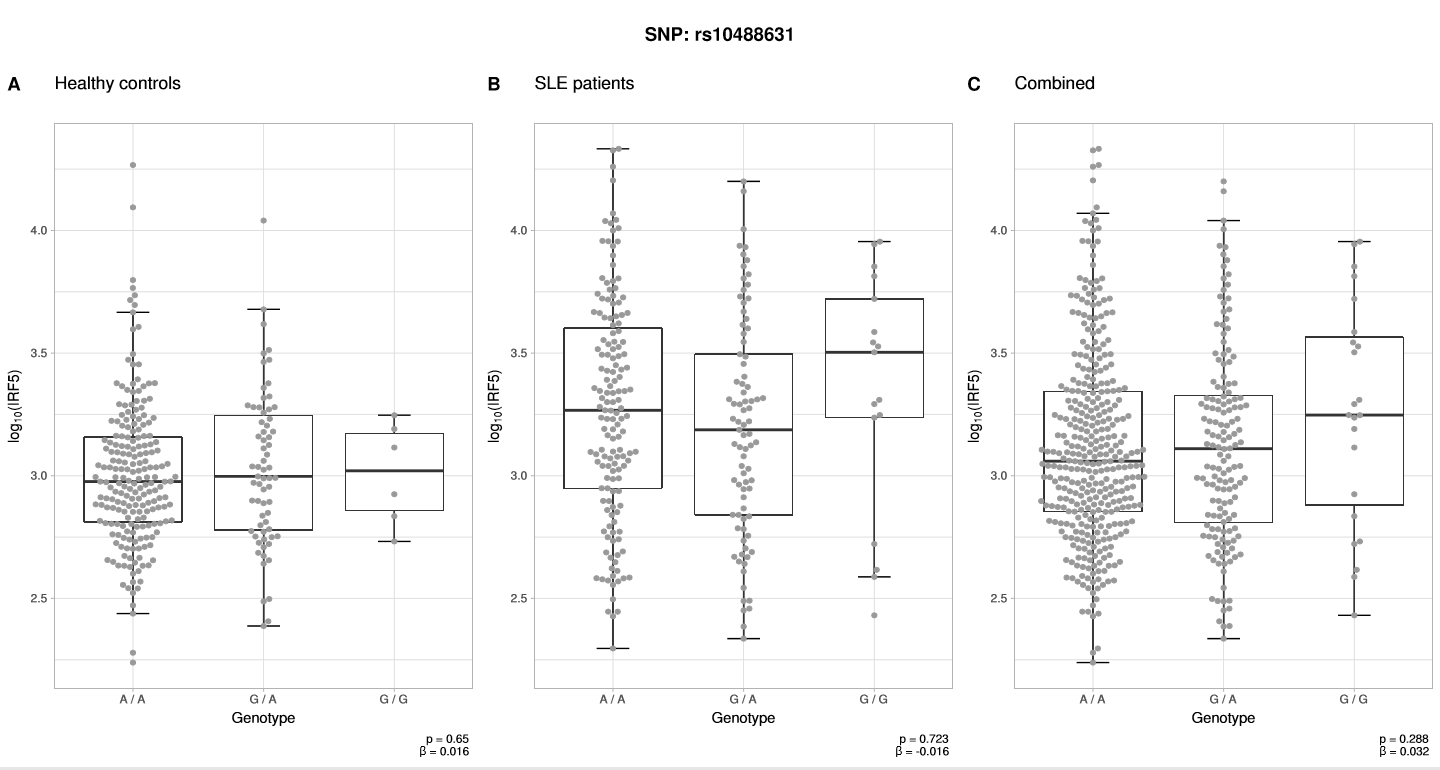


**Supplementary Figure S-8**. Associations between log_10_ IRF5 protein levels and the *IRF5* SLE risk variant rs10488631 are shown for A) healthy controls, B) SLE patients and C) both SLE patients and healthy controls combined.

**References**

1. Checa A, Idborg H, Zandian A, Sar DG, Surowiec I, Trygg J, et al. Dysregulations in circulating sphingolipids associate with disease activity indices in female patients with systemic lupus erythematosus: a cross-sectional study. *Lupus* (2017):096120331668670. doi: 10.1177/0961203316686707.
