# Supplementary material for: Circulating Levels of Interferon Regulatory Factor-5 Associates With Subgroups of Systemic Lupus Erythematosus Patients
Source: Front Immunol. 2019 May 17;10:1029. doi: 10.3389/fimmu.2019.01029 (PMC6533644; doi:10.3389/fimmu.2019.01029)
Supplement: Supplementary file 3 [file Data_Sheet_3.docx]

**Supplementary information Methods and Results**

***Recombinant protein (IRF5) production, method details and results***

Multiple constructs of IRF5 (Uniprot ID Q13568) were sub-cloned into the expression vectors pNIC28-Bsa4 and pNIC-Bio3 (Genbank acc. no code EF198106, JN792439), which both carries an N-terminal His6-tag followed by a TEV protease cleavage site. In addition, pNIC-Bio3 carries a C-terminal Avi-tag for site-specific biotinylation. The pNIC-Bio3 expression plasmids were transformed into E. coli expression strain BL21(DE3) R3 pRARE2 carrying a plasmid for co-expression of BirA ligase (pCDF-BirA). After performing small-scale screening for soluble recombinant protein expression as previously described (22) clones corresponding to constructs covering regions M1-V120 and E232-L434 were selected to be used as antigens in phage display generation of single-chain fragment variable (scFv) binders.

For production of biotinylated antigen, selected clones were grown at 37°C to an OD600 of 1.5–2.0 in Terrific broth (TB) medium supplemented with Kanamycin (50 µg/mL), Chloramphenicol (35 µg/mL), Spectinomycin (50 µg/mL) and Biotin (100 µM). After lowering the temperature to 18°C, protein expression was induced by addition of isopropyl-β-D-thiogalactopyranoside to a final concentration of 0.5 mM. Cultures were incubated for ~20 hours and then harvested by centrifugation at 4500 × g for 15 minutes and re-suspended in buffer containing 50 mM HEPES pH 8.0, 500 mM NaCl, 5% glycerol, 10 mM imidazole, 0.5 mM TCEP and Complete EDTA-free protease inhibitor (Roche). Re-suspended cells were sonicated on ice followed by centrifugation at 44000 × g for 50 minutes. The soluble fraction was decanted, filtered (0.45 µm) and subsequently loaded onto a HiTrap Ni-chelating column (GE Healthcare) on an ÄKTA Xpress (GE Healthcare). After washing with 20 mM HEPES pH 7.5, 500 mM NaCl, 5% glycerol, 10 mM imidazole and 0.5 mM TCEP, the protein was eluted in 20 mM HEPES pH 7.5, 500 mM NaCl, 5% glycerol, 250 mM imidazole and 0.5 mM TCEP. The eluate was applied to a Hiload XK16/60 Superdex 200 column (GE Healthcare) equilibrated with 20 mM HEPES, pH 7.5, 300 mM NaCl, and 0.5 mM TCEP. Relevant SEC fractions were pooled and analyzed by SDS-PAGE and mass spectrometry to assess quality and verify protein identity.

An expression plasmid containing full-length IRF5 in vector pNIC28-Bsa4 was used for production of tag free samples for experiments. Protein production was performed following the same protocol outlined above excluding co-expression with BirA and Biotin. In addition, purified IRF5 was subjected to treatment with His6-TEV protease for removal of the N-terminal His6-tag (~ 1:50 ratio of TEV protease:substrate; w/w). After completion of the reaction, an additional step of Ni2+-affinity purification using Ni-NTA agarose resin (GE Healthcare) was performed to separate cleaved IRF5 from any remaining His6-tagged IRF5 and the TEV protease. All protein batches were flash frozen in liquid nitrogen and stored at -80°C until use.

***Detection of IRF5-positive microparticles***

*Samples subjected to analysis of IRF5 positive microparticles in plasma:*

63 SLE patients that fulfilled at least four of the 1982 revised classification criteria for SLE according to the American College of Rheumatology were selected (1).. The patients were between 17 and 81 years of age (median 45 years) and 86% were females. 20 healthy control samples with matching mean age and gender were also included.

Blood sampling was performed after overnight fasting according to standardized protocols. Blood was drawn into vacutainer tubes containing sodium citrate (0.129 M). Platelet-poor plasma (PPP) was obtained after centrifugation at 2570 9 g for 20 min at room temperature (RT), and frozen within 1 h at -80 °C until analysis.

*Measurement of MPs*

PPP was thawed in a water bath for roughly 5 min (37 °C), and then centrifuged initially at 2000g for 20 min at RT. The supernatant was then re-centrifuged at 13 000g for 2 min at RT. Subsequently, 20 µL of the supernatant was incubated for 20 min in the dark with 5 µl, polyclonal anti-IRF5–FITC (Biorbyt, Cambridge, UK). According the company, this antibody has been validated in assays with the addition of purified IRF5 protein. In order to phenotype IRF5+ MPs, antibodies towards platelet origin (CD42a, 5µl; Abcam, Cambridge, UK), Leukocyte origin (CD45, 5µl; Abcam, Cambridge, UK) and endothelial origin (CD62E, 5µl; Abcam, Cambridge, UK) were used. MPs were measured by flow cytometry on a Beckman Gallios instrument (Beckman Coulter, Bream CA, USA). The MP gate was determined by the use of Megamix-Plus FSC beads (0.1, 0.3, 0.5 and 0.9 µm in size; BioCytex, Marseille, France). MPs were defined as particles between ~ 0.3 µm and 0.9 µm in size (forward scatter). Conjugate isotype matched immunoglobulin (FITC) with no reactivity against human antigens were used as negative controls. MP results are presented as MPs/µl plasma. The intra-assay and inter-assay coefficients of variation for MP measurement were < 9%.

To evaluate if anti-IRF5-FITC binds to MPs the following experiment was performed. MPs in a SLE plasma sample were labeled with anti-IRF5 as described above and detected by flow cytometry. As a control sample, MPs from same SLE sample were depleted by high speed centrifugation (20 800g for 60 min at room temperature). Results demonstrate that the majority of the IRF5 are exposed on particles (Figure S-M1).

**
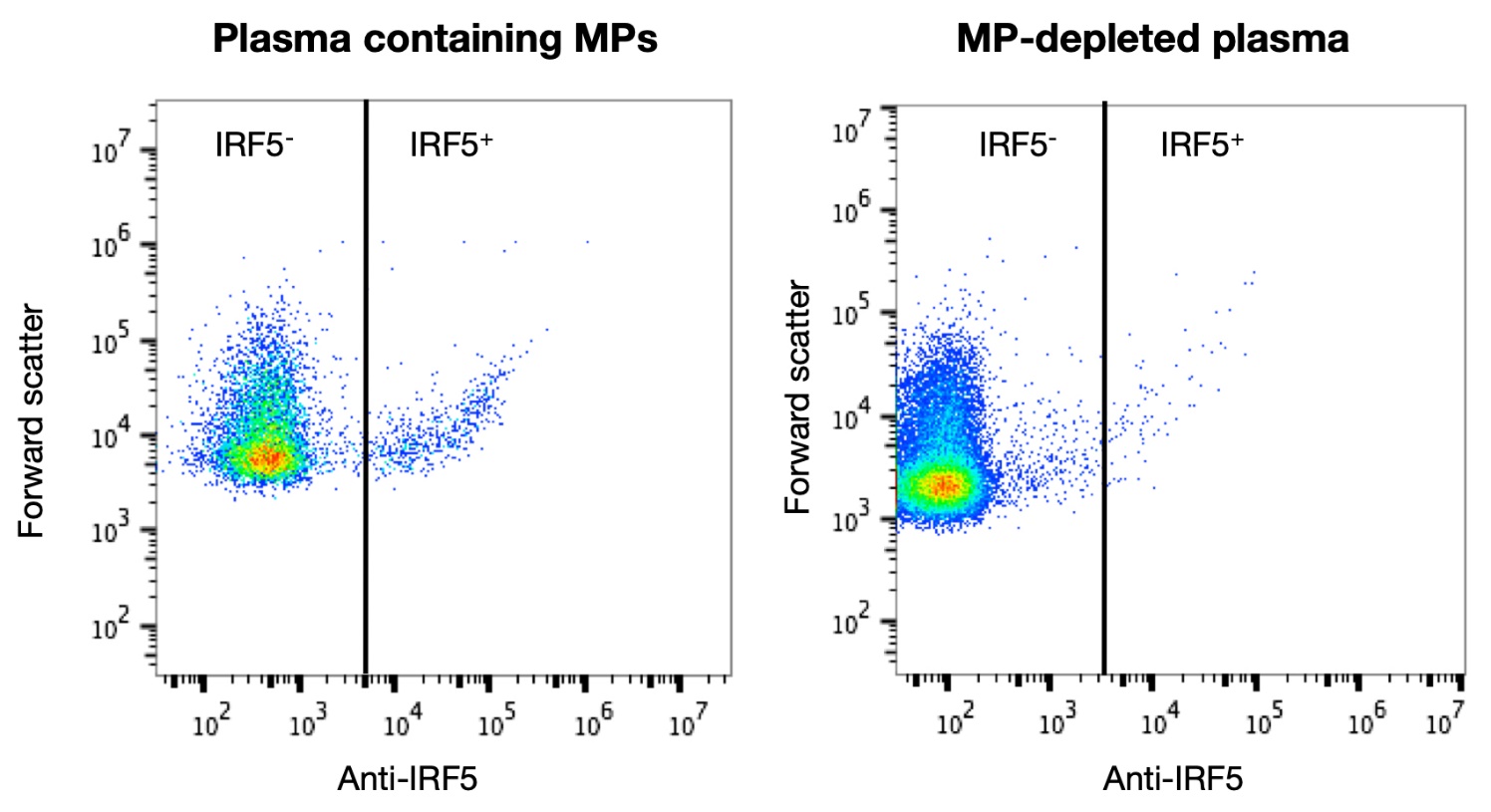
**

*Figure S-M1. Representative figure demonstrating IRF5 exposure on MPs and MP-depleted plasma in a SLE patient.*
